# Carotenoid and Carotenoid Ester Profile and Their Deposition in Plastids in Fruits of New Papaya (*Carica papaya* L.) Varieties from the Canary Islands

**DOI:** 10.3390/foods10020434

**Published:** 2021-02-17

**Authors:** Sara Lara-Abia, Gloria Lobo-Rodrigo, Jorge Welti-Chanes, M. Pilar Cano

**Affiliations:** 1 Department of Biotechnology and Food Microbiology, Institute of Food Science Research (CIAL) (CSIC-UAM), 28001 Madrid, Spain; sara.lara.abia@gmail.com; 2 School of Sciences and Engineering, Tecnológico de Monterrey (ITESM), Monterrey 64000, Mexico; jwelti@tec.mx; 3 Department of Crop Production in Tropical and Subtropical Areas, Instituto Canario de Investigaciones Agrarias (ICIA), 38270 Tenerife, Spain; globo@icia.es

**Keywords:** *Carica papaya* L., carotenoids, carotenoid esters, HPLC-PDA-MS (APCI^+^), carotenoid deposition, microstructure, plastids

## Abstract

The carotenoid profile of non-saponified and saponified extracts of different tissues (pulp and peel) of fruits of three new papaya varieties, Sweet Mary, Alicia, and Eksotika, was characterized for the first time, and almost all carotenoid compounds were quantified. Carotenoids and carotenoid esters were analyzed and characterized using HPLC-photo diode array (PDA-MS with atmospheric pressure chemical ionization with positive ion mode (APCI^+^) with a C_30_ reversed-phase column. The carotenoid deposition in collenchyma and chlorenchyma cells of papaya pulp and peel tissues was assessed by optical microscopy, confocal laser scanning microscopy, and transmission electron microscopy. The most abundant carotenoids in the fruit of the three papaya varieties (pulp and peel) were (all-*E*)-lycopene (230.0–421.2 µg/100 g fresh weight), (all-*E*)-β-carotene (120.3–233.2 µg/100 g fresh weight), and (all-*E*)-β-cryptoxanthin laurate (74.4–223.2 µg/100 g fresh weight. Moreover, high concentrations of (all-*E*)-lutein (922.5–1381.1 µg/100 g fresh weight) and its esters, such as (all-*E*)-lutein-3-*O*-myristate and (all-*E*)-lutein dimyristate, were found in peel extracts. The optical microscopy study of papaya pulps showed that carotenoid deposition in all papaya varieties, including Maradol, was mainly localized close to the cell walls, showing the presence of some crystalloids and round-shaped structures, with different sizes and distribution due to the different carotenoid content among varieties. No crystalloids or globular depositions were found in any of the peel sections, and no remarkable differences were found in the papaya peel microstructure of the different papaya varieties.

## 1. Introduction

Papaya (*Carica papaya* L.) belongs to the Caricaceae family and it originates from South America. Papaya fruits represent 10% of the world production of tropical fruits. The main papaya-producing countries are India (5.5 million tons), Brazil (1.6 million tons), Indonesia (0.9 million tons), and Mexico (0.8 million tons) [1]. In Spain, papaya cultivation is concentrated mostly in the Canary Islands where it has increased in the last few years from 138 ha in 2004 to 350 ha in 2016, with an annual production of 16,000 tons per year [2]. Recently, new papaya varieties were introduced to the market: Sweet Mary, Alicia, and Eksotika. These varieties are originally from Mexico and they are interesting to Spanish growers and farmers due to their physical characteristics, considering the preferences of the European market: (1) homogeneous production, (2) low height on the stem, (3) medium size, (4) pear shape, and (5) sweet fruits [3,4].

Until now, scientific research based on the characterization of Sweet Mary, Alicia, and Eksotika papaya varieties has been scarce. Most of the studies focusing on carotenoid characterization in papaya were performed with the Maradol variety and usually in saponified extracts [5]. There are some published papers related to the growing conditions of these papaya varieties in Canary Islands (Spain). For instance, Cabrera et al. [2] reviewed the conditions inside two papaya (cv. Sweet Mary) greenhouses, located in different regions of Tenerife Island, comparing their effect on yield and fruit quality along the commercial crop life. Addai et al. [6] evaluated the effect of the maturity stage of papaya cv. Eksotika, in terms of its physicochemical properties, antioxidant capacity, and sensory characteristics. With respect to the carotenoid composition of papaya fruits, Sancho et al. [7] characterized the phenolic compounds in papaya peel and the main carotenoids found in papaya Maradol pulp extracts. These researchers provide a preliminary approach to carotenoid characterization using HPLC-DAD, identifying and quantifying only β-cryptoxanthin, β-carotene, and lycopene in saponified pulp extracts. Furthermore, Cano et al. [8] reported the composition in carotenoids of saponified extracts of hermaphrodite and female papaya fruits (*Carica papaya* L.) cv. Sunrise from Canary Islands (Spain) during post-harvest ripening. However, further carotenoid and carotenoid ester characterization and quantification, in both pulp and peel, are needed to have a complete composition of these important bioactive compounds in papaya tissues.

Carotenoids are tetraterpenoids are composed of eight isoprene units. Considering the chemical elements in their structure, carotenoids are classified into two groups, carotenes (hydrocarbons) and xanthophylls (containing oxygen). In fruits and vegetables, xanthophylls may be found either in a free unesterified form, esterified to saturated fatty acids such as lauric (C12:0), myristic (C14:0), palmitic (C16:0), and stearic (18:0) acids [9], or esterified to unsaturated fatty acids such as linoleic (C18:2) or linolenic (C18:3) acids, forming carotenoid esters [10].

Since most of the fruit and vegetables are rich in xanthophyll esters, recent investigations have more been focused on the study of non-saponified carotenoid-rich extracts in a manner that enables the evaluation of the complete native carotenoid composition of plant tissues [10]. High quantities of carotenoid esters can be found in common fruits such as apple (*Malus x domestica*) [11], sweet orange (*Citrus sinensis* L.) [12], mango (*Mangifera indica* L.) [13], and mandarin (*Citrus reticulata* L.) [14], as well as in less common fruits, such as persimmon (*Diospyros kaki* Thunb.) [15], cashew apple (*Anacardium occidentale* L.) [16], and lucuma (*Pouteria lucuma* (Ruiz & Pav.) Kuntze) [17].

The aim of this study was to describe the complete carotenoid profile present in Sweet Mary, Alicia, and Eksotika papaya (*Carica papaya* L.) varieties, and to conduct the quantification of carotenoid species present in different tissues (pulp and peel) of these papaya varieties. In addition, microstructural studies of the three varieties were done as complementary information, due to the fact that the bioavailability of carotenoids is related to the microstructure of fruit tissue cells and carotenoid deposition [18]. Moreover, a comparison with papaya cv. Maradol originally from Mexico was conducted. This study reveals for the first time the complete carotenoid and carotenoid ester profile in papaya fruits in non-saponified (direct) extracts and their deposition structures in mature fruits. We expect to contribute to the exploration of new papaya varieties as interesting carotenoid sources and to provide new knowledge about the healthy potential of this tropical fruit for consumption (edible pulp) or to obtain ingredients or nutraceuticals from industrial papaya byproducts such as fruit peels.

## 2. Materials and Methods

### 2.1. Chemicals and Standards

Methanol (MeOH), diethyl ether, tetrahydrofuran (THF), methyl *tert*-butyl ether (MTBE), and acetone were purchased from VWR International (Radnor, Pensilvania, USA); ultrapure water was obtained from a Millipak^®^ Express 40 system (Merk-Millipore, Darmstadt, Germany); anhydrous sodium sulfate, potassium hydroxide (KOH), and sodium chloride (NaCl) were purchased from Panreac Quimica (Barcelona, Spain); butylated hydroxytoluene (BHT) and magnesium carbonate were obtained from Acros Organics (New Jersey, USA). Standards for lycopene (L9879, ≥90%, from tomato), lutein (X6250 from marigold), and (all-*E*)-β-apo-8’-carotenal (10810, ≥96%, (ultraviolet (UV))) were purchased from Sigma-Aldrich (St. Louis, Missouri, USA). Standards for (all-*E*)-β-carotene (HPLC 96%, synth., cryst.), (all-*E*)-α-carotene (HPLC 97%, synth., cryst.), (all-*E*)-β-cryptoxanthin (HPLC 97%, synth., cryst.), (all-*E*)-zeaxanthin (HPLC 97%, synth., cryst.), (all-*E*)-neoxanthin (HPLC 97%, isolated, cryst.), and (all-*E*)-violaxanthin (HPLC 95%, isolated, cryst.) were from CaroteNature (Ostermundigen, Switzerland). Reagents used for microscopy (neutral red (N7005, ≥90% and calcofluor white stain) were obtained from Sigma-Aldrich (St Louis, MO, USA).

### 2.2. Papaya Fruits

Mature papayas (*Carica papaya* L.) Sweet Mary, Alicia, and Eksotika varieties were grown using hydroponic systems in greenhouses in Güimar borough (Santa Cruz de Tenerife, Canary Islands, Spain: 28° 18’ 52” north (N); 16° 24’ 36” west (W); 271 m above sea level) and, after, harvest fruits of the three varieties were air-transported to the Institute of Food Science Research (CIAL, CSIC-UAM) laboratory in Madrid. Maradol papaya fruits originally from Mexico were purchased from a local market in Madrid (Spain). Fruits were washed and selected according to uniform maturity, size, and no defects. Maturity level 5 was chosen for all papaya fruits following the ripening stages reported by Ramos-Parra et al. [19].

The physical and physicochemical characteristics of papaya fruits (Table 1) were evaluated as described before [20]. The physicochemical characteristics such as apical caliber (cm), equatorial caliber (cm), and weight (g) were determined directly in 10 whole fruits of each variety (Table 1). Titratable acidity (g citric acid/100 g fresh weight) was determined by neutralization of papaya pulp juice with 0.1 N sodium hydroxide until a pH value of 8.1 pH and soluble solids (°Brix at 25 °C) were also measured from juice obtained from papaya pulps. Color of pulps and peels was recorded using the L* (lightness), a* (green–red tonality), b* (blue–yellow tonality) scale CIELAB system with a Konica Minolta CM-3500d (Japan).

After washing and draining, papaya fruits were cut, seeds were manually removed, and two types of tissue, pulp and peel (less than 2 mm), were separated. Pieces (20 × 20 mm) of each papaya tissue were vacuum-packaged in 200 × 300 mm plastic bags (Cryovac^®^, Sealed Air Corporation, Madrid, Spain), frozen with liquid nitrogen, and freeze-dried for 5 days at −45 °C and 1.3 × 10^−3^ MPa (LyoBeta 15, Azbil Telstar SL, Terrasa, Spain). Freeze-dried material was ground by pulverizing (Grindomix GM200, Retsch, Germany) to a fine particle size (<2 mm), vacuum-packed in plastic bags, and stored at −80 °C until carotenoid analysis.

### 2.3. Carotenoid Extraction and Saponification

#### 2.3.1. Carotenoid Extraction from Papaya Tissues

The extraction of carotenoids and carotenoid esters was performed according to Cano et al. [15] with some modifications. Extraction and saponification procedures were carried out under dim light, using amber flasks and avoiding long-term oxygen exposure. First, 1 g of freeze-dried sample was mixed with 0.5 g of magnesium carbonate and 60 μL of (all-*E*)-β-apo-8’-carotenal (0.40 mg/mL), as an internal standard. Then, 20 mL of tetrahydrofuran (THF) stabilized with 0.01% (*w/v*) butylated hydroxytoluene (BHT) was added for the extraction. The sample was homogenized in an Omnimixer (OMNI Macro S^®^, OMNI International, Kennesaw, USA) for 3 min at 7000 rpm and placed in an ultrasonic water bath (3000514 model, 50/60 Hz, 360 W, J. P. Selecta S.A., Barcelona, Spain) for 30 min. The extract was centrifuged at 7000× *g* for 10 min at 4 °C, and the supernatant was collected. After, 20 mL of acetone was added to the pellet, and the sample was extracted again. The supernatants were combined and placed in a separation funnel, adding e 20 mL of diethyl ether. The funnel was shaken to properly mix all the volumes inside, and the organic phase was collected in a round amber flask. If an emulsion was formed, 10 mL of ultrapure water was added to improve the separation of the organic and inorganic phases. The organic phase was collected and dried with 2.5 g of anhydrous sodium sulfate for 10 min at room temperature. Then, the sample was filtered through Whatman No. 1 filter paper. The sample was dried at 30 °C in a rotatory evaporator, dissolved to exactly 2 mL with MeOH/MTBE/H_2_O (45.5:52.5:2, *v/v/v*), filtered through a 0.45 μm filter, and analyzed by HPLC.

#### 2.3.2. Saponification of Carotenoid Extracts

First, 4 mL of 30% methanolic potassium hydroxide (KOH) was added to the dried organic phase containing carotenoids and kept under magnetic agitation for 1.5 h in a nitrogen atmosphere in the dark. After that, the saponified extract was added to a funnel containing 15 mL of diethyl ether and was washed five times with 20 mL of ultrapure water saturated with 30% (*w/v*) NaCl, removing the aqueous phase each time, until reaching neutral pH. The extract was dried with anhydrous sodium sulfate, filtered, and drained on a rotatory evaporator with controlled temperature (30 °C). The residue was made up to 2 mL with MeOH/MTBE/H_2_O (45.5:52.5:2, *v/v/v*); lastly, this solution was filtered through a 0.45 μm filter and immediately analyzed by HPLC.

### 2.4. Carotenoid Analysis by HPLC with Diode Array Detector (DAD)

The identification and the quantification of carotenoids and carotenoid esters in saponified and non-saponified papaya extracts from peel and pulp tissues were performed according to the methodology reported by Cano et al. [15] using a 1200 Series Agilent HPLC System (Agilent Technologies, Santa Clara, CA, USA) with a reversed-phase C_30_ column (YMC-Pack YMC C_30_, 250 × 4.6 mm inner diameter (i.d.), S-5 μm, YMC Co., Ltd., Kyoto, Japan). The mobile phases used for the separation was MeOH/MTBE/H2O (81:14:4, *v/v/v*, eluent A) and MeOH/MTBE (10:90, *v/v*, eluent B), both containing 0.1% ammonium acetate. The elution gradient was linear, starting at 100% A and ending with 100% B, in 60 min. The flow rate was 1 mL/min, the column temperature was 32 °C and the injection volume was 20 μL; the injection chamber was set at 4 °C to avoid instability of carotenoids. Free and esterified carotenoids were monitored at 450 nm and additional UV/visible light (Vis) spectra were recorded in the range of 220–700 nm.

The identification of carotenoids was accomplished according to their elution time, chromatography with carotenoid standards, UV-visible spectrum (λ_max_, spectral fine structure (%III/II), peak *cis* intensity), and mass spectrum (see Section 2.5) compared with available data [15,21,22,23,24,25,26].

Carotenoid quantification was performed using linear calibration curves prepared with concentrations in the range of 5–100 μg/mL of carotenoid stock solutions. The (all-*E*)-β-carotene curve was used for quantifying β-carotene and β-carotene isomers, and the (all-*E*)-α-carotene curve was used for quantifying α-carotene and α-carotene isomers. The (all-*E*)-lutein calibration curve was used for lutein-epoxide and lutein ester quantification. Contents of β-cryptoxanthin esters and α-cryptoxanthin were calculated on the basis of the (all-*E*)-β-cryptoxanthin curve. The (all-*E*)-violaxanthin standard curve was used to quantifie the violaxanthin isomers and (all-*E*)-antheranxanthin. Carotenoids such as (all-*E*)-neoxanthin and (all-*E*)-lycopene were quantitated by their corresponding standards. Results were expressed as micrograms of the corresponding carotenoid per 100 g of fresh weight. Carotenoid esters were quantified using the calibration curves of their corresponding carotenoids. Vitamin A value was calculated as retinol activity equivalent (RAE) per 100 g of fresh weight, following the equation RAE = (μg of β-carotene/12) + (μg of other pro-vitamin A carotenoids (such as β-cryptoxanthin and β-cryptoxanthin esters)/24) [27].

### 2.5. Liquid Chromatography-Mass Spectrometry (LC-MS/MS)

LC-MS/MS using atmospheric pressure chemical ionization with positive ion mode (APCI^+^) analyses was performed with the same HPLC system described previously. It was coupled online to an Agilent mass spectrometry detector with APCI source model G1947B compatible with the LCMS SQ 6120 equipment, according to the procedure described by Breithaupt and Schwack [28]. The positive ion mass spectra of the column eluted at 13,000 Th/s (peak width 0.6 Th, full width at half maximum FWHM)). Nitrogen was used as the drying gas at a flow rate of 60 L/min and as the nebulizing gas at a pressure of 50 psi. The nebulizer temperature was set at 350 °C, and a potential of +2779 kV was used on the capillary. The corona was set at 4000 nA in positive ion mode, and the vaporizer temperature was 400 °C. The collision gas was helium, and the fragmentation amplitude was 0.81.2 V. The chromatographic conditions were the same as described for the quantitative analyses of carotenoids.

### 2.6. Microscopy Analysis of Papaya Tissues

#### 2.6.1. Optical Light Microscopy

For light microscopy observation, cryostat sections (20 μm) were obtained from papaya pulp cubes. They were frozen at −80 °C in an ultra-low-temperature freezer, fixed with ethanol 95%, and placed in a paraffin embedding station (Leica EG1160). Then, the samples were transferred to a cryostat (Leica CM1900) and mounted on a slide using a standard motorized microtome (Leica RM2155). To observe carotenoid compounds, no dye was used to stain the samples. Light microscopy was performed with a vertical microscope Axioskop (Carl Zeiss, Germany) coupled to a Leica DMC 6200 pixel shift camera (Leica Microsystems, Germany). Samples were observed with an open condenser, level 4 of illumination, and no color filters were used. The color was manually adjusted to show real-time colors (approximately 66% brightness, 46% saturation, and 0.80 gamma) using Leica Application Suite software. Samples were observed at 20× and 40× with a Zeiss Plan-Neofluar lens with the addition of an immersion oil drop. Three replicas of each sample were prepared and analyzed.

#### 2.6.2. Confocal Laser Scanning Microscopy

Microstructural analysis was also performed using confocal laser scanning microscopy (CLSM). To observe cell walls in papaya fruit and their possible autofluorescence, microtome-cut sections of papaya pulp were placed on a slide and stained with 0.1% calcofluor white M2R (*w/v*) solution during 5 min. The excitation and emission of the dye were measured at 405 and 430 nm, respectively. To examine the presence of autofluorescence in papaya cells, unstained sections were observed through a broad range (405–633) of excitation and band-pass emission filter. This analysis was achieved using a Confocal multispectral TCS SP8 system (Leica Microsystems, Germany) at 20× and 40× with a Zeiss Plan-Neofluar lens. At least three replicas of each sample were prepared and analyzed.

#### 2.6.3. Transmission Electron Microscopy

Transmission electron microscopy (TEM) was used to observe microstructural differences between Sweet Mary, Alicia, and Eksotika papaya varieties from the Canary Islands and papaya cv. Maradol from Mexico. Moreover, it was used to observe organelles and cell organization in papaya tissues (pulp and peel). Fresh papaya tissues sections (1 mm) were hand-cut. Pulp and peel sections were placed in a fixing solution of 3% glutaraldehyde in Phosphate-buffered saline (PBS) buffer (pH 7.4) for 1 h at room temperature. Afterward, samples were post-fixed in 1% osmium tetroxide in PBS for 1 h at 4 °C. They were dehydrated with a graded series of ethanol (30–100%) and embedded in a low-viscosity epoxy resin (SPURR). The resin was hardened for 48 h at 60 °C. Then, the preparations were cut using a Reichert-Jung ultra-cut ultra-microtome (Leica Microsystems, Germany) and mounted on a collodion carbon-coated grid. Ultrathin sections (60 nm) were stained with uranyl acetate and lead citrate. Lastly, samples were analyzed using a Jem 1230 electron microscope (JEOL Ltd., Germany) with an accelerating voltage of up to 120 kV and captured with digital with image sensors type CMOS Camera Tem-Cam (TVIPS GmbH, Germany). Three replicas of each sample were prepared and analyzed.

### 2.7. Statistical Analysis

Results were expressed as the mean ± standard deviation (*n* = 4). This came from obtaining at least two independent extracts (*n* = 2) and by performing the determinations of each two times (*n* = 2). Significant differences (*p* < 0.05) were calculated by one-way analysis of variance (ANOVA), followed by a post hoc Tukey’s test. Statistical analyses were performed with IBM^®^ SPSS^®^ Statistics 23.0 (IBM Corp, Armonk, NY, USA).

## 3. Results and Discussion

### 3.1. Fruits of Papaya: Origin and Comparison

The fruits of the three new varieties of papaya collected in the Canary Islands, Sweet Mary, Alicia, and Eksotika, and the fruits of the papaya of the Maradol variety of Mexican origin were obtained on the same date, in June 2019. Papayas of Canarian origin were transported by air to the CIAL (CSIC-UAM) laboratories in Madrid (Spain), and fruits of papayas cv. Maradol were acquired at a local supermarket, with their Mexican origin verified by the suppliers (see Section 2). All papaya fruits were selected and stored at the appropriate temperature (21 °C) until they reached ripening stage 5 (full maturity) [19] for the conduct of this study. Therefore, representative fruit samples (no less than 12 fruits) of each variety of papaya were selected so that the variability that could come from the differences in maturity or origin of the fruit was minimized. However, it must be borne in mind that the agronomic conditions for papaya fruit production in Mexico and in Spain may not be the same. The physical and physicochemical characteristics of the studied papaya fruits are showed in Table 1.

### 3.2. Characterization of Carotenoid and Carotenoid Ester Profile of Papaya Fruits

**Figure 1:**
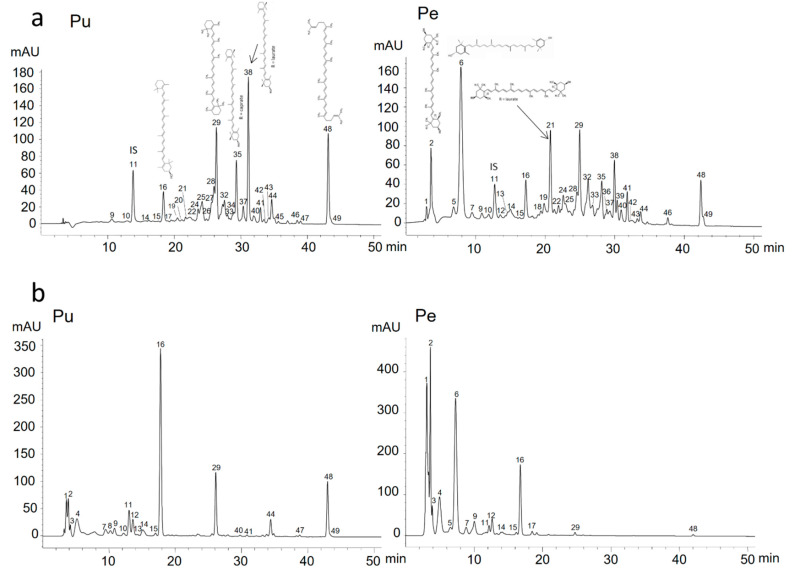
C_30_ reversed-phase chromatogram of carotenoids of direct (**a**) or saponified extracts (**b**) obtained from pulp (PU) and peel (PE) of papaya fruits (*Carica papaya* L.) cv. Sweet Mary, detected at 450 nm. Peak identities are shown in Table 2.

Carotenoids of the three papaya varieties from the Canary Islands and Maradol papaya fruits were identified on the basis of the combined information obtained from chromatographic elution, UV/visible data, and mass spectral characteristics. Additionally, MS experiments confirmed the assignment of the protonated molecule ([M + H]^+^) of all identified peaks through the fragments expected for the carotenoid polyene chain and functional groups (Table 2). A detailed description of carotenoid identification in the HPLC profile of each of the new papaya varieties, Sweet Mary, Alicia, and Eksotika, and the Maradol variety, taking into account some of the most important spectral characteristics of each carotenoid, are discussed below. Figure 1 (cv. Sweet Mary), and Figure S1 (cv. Alicia), Figure S2 (cv. Eksotika), and Figure S3 (cv. Maradol) (Supplementary Materials) show the HPLC chromatograms obtained from the different studied papaya varieties of direct (non-saponifed) (A) and saponified extracts of both (B) pulp (edible part) and peel (byproduct) tissues analyzed using HPLC-DAD on a C_30_ column.

The identification of carotenoids is presented in Table 2. Carotenoid profiles revealed a great qualitative diversity of carotenoids in the studied papaya varieties, composed of 48 carotenoids; we identified 16 free xanthophylls, 15 hydrocarbon carotenoids, and 15 xanthophyll esters. Table 2 also shows two more peaks, which were not completely identified.

#### 3.2.1. Free Xanthophylls in Papaya Fruits

Peak 1 (retention time (Rt) = 3.4 min) and peak 2 (Rt = 3.8 min) were identified as (13*Z*)-violaxanthin and (all-*E*)-violaxanthin, respectively. The UV/Vis spectrum of peak 1, with λ_max_ at 416, 440, and 470 nm showed the appearance of a “*cis* peak” at 326 nm (%III/II = 78). LC/MS (APCI^+^) spectra of peaks 1 and 2 showed a protonated molecule [M + H]^+^ at *m*/*z* 601 which was consistent with the molecular formula C_40_H_56_O_4_ (molecular weight (Mw) = 601.426 g/mol), whereas the authentic standard also presented these [M + H]^+^ values. Similar characteristics showed the carotenoid compound of peak 5 (Rt = 7.6 min) identified as (all-*E*)-neoxanthin (λ_max_ at 416, 437, and 469 nm). Peak 4 (Rt = 5.2 min) was identified as (9*Z*)-neoxanthin (λ_max_ at 327, 410, 432, and 462 nm), which could have been produced via isomerization during the saponification process.

Peaks 1 and 4 showed higher concentrations in papaya saponified extracts (Figure 1; Figures S1 and S2, Supplementary Materials), indicating their main production during the saponification process. These two peaks are “*cis*-isomers” of violaxanthin and neoxanthin.

Peak 6 (Rt = 7.9 min) was identified as (all-*E*)-lutein, showing a UV/Vis spectrum with λ_max_ at 420, 444, and 472 nm (%III/II = 62). Its identification was also made via comparison with a commercial pure standard lutein sample. Peak 7 (Rt = 9.5 min) was identified as (all-*E*)-zeaxanthin, considering UV/Vis (λ_max_ at 426, 450, and 470 nm) and mass spectral characteristics and it was confirmed by co-elution with the (all-*E*)-zeaxanthin standard. Lutein-5,6-epoxide (peak 8; Rt = 10.3 min; λ_max_ at 418, 440, and 468 nm) and (all-*E*)-antheraxanthin (peak 9; Rt = 10.9 min; λ_max_ at 422, 444, and 472 nm) were identified according to their spectroscopic characteristics and the available literature [9]. Peak 10 (Rt = 12.3 min) was tentatively identified as (9*Z*)-violaxanthin, showing UV/Vis and mass spectral features similar to those reported in the literature [22,23]. Peak 11 (Rt = 13.2 min) was clearly (all-*E*)-β-apo-8’-carotenal, added to the extracts to evaluate the recovery of carotenoids in the extraction process. Its UV/Vis and mass spectral characteristics are shown in Table 2. Peak 12 (Rt = 13.7 min) was pointed out as β-cryptoxanthin-5, 6-epoxide, according to its spectroscopic characteristics, with λ_max_ at 420, 445, and 471 nm (%III/II = 52), and to its MS spectrum. To distinguish between (all-*E*)-α-cryptoxanthin (peak 15) and (all-*E*)-β-cryptoxanthin (peak 16), the identification was based on the HPLC co-elution with authentic commercial standards for both. Furthermore, the presence of these peaks was confirmed with the available literature [31].

Peaks 22 (Rt = 22.1 min) and 24 (Rt = 23.4 min) were tentatively identified as β-cryptoxanthin-5,8-epoxide and β-cryptoxanthin-5,8’-epoxide, respectively. The identification was established by comparing its UV/Vis spectrum and retention time with the literature reported by De Rosso and Mercadante [32].

Previous studies published by Chandrika et al. [33] and Sancho et al. [7] reported the identification and quantification of carotenoids in papaya cv. Maradol. The singularity of these investigations remains in the simple mention of β-cryptoxanthin as the sole free xanthophyll present in papaya pulp. In the present investigation, 15 free xanthophylls are described for the first time in papaya pulp and peel.

#### 3.2.2. Hydrocarbon Carotenes in Papaya Fruits

The first hydrocarbon carotenes to elute were α-carotene-5,6-epoxide (peak 17; Rt = 19.3 min; λ_max_ at 418, 441, and 469 nm), (13*Z*)-α-carotene (peak 19; Rt = 20.3 min; λ_max_ at 413, 439, and 467 nm), and (13*Z*)-β-carotene (peak 20; Rt = 21.2 min; λ_max_ at 414, 436, and 464 nm). The UV absorption spectra of the (13*Z*)-isomers showed a *cis* peak at 337 nm.

(all-*E*)-ζ-Carotene (peak 23; Rt = 22.5 min) was identified considering its λ_max_ at 378, 400, and 423 nm and high fine structure in the UV/Vis spectrum. The protonated molecule was detected at *m*/*z* 541, and the MS/MS showed the presence of fragments at *m*/*z* 472 and 404, formed by bis-allylic cleavage between C-3 and C-4 [M + H − 69]^+^ and between C-7 and C-8 [M + H − 137]^+^, also confirmed with the literature [9,23,34].

Peak 25 (Rt = 24.0 min) (all-*E*)-α-carotene showed the same chromophore as lutein; thus, the UV/Vis absorption spectrum was similar in both of them. The identification was confirmed through chromatographic behavior, co-elution with (all-*E*)-α-carotene standard, and mass spectrum.

(all-*E*)-β-Carotene (peak 29; Rt = 26.2 min) was identified by comparison with its corresponding standard. The most relevant characteristic that helps to distinguish the all-*trans* isomers of α-carotene and β-carotene is the presence of the α-ionone moiety of *m*/*z* 123, observed in the positive ion APCI (^+^) mass spectrum of (all-*E*)-α-carotene but not in (all-*E*)-β-carotene [26]. Peak 26 (Rt = 24.7 min) and peak 30 (Rt = 26.8 min) were described as (9*Z*)-α-carotene and (9*Z*)-β-carotene, respectively. The elution order of carotene isomers reported previously in the literature [30] showed a first elution of (13*Z*), followed by (all-*E*) and (9*Z*). However, in the present study it was seen that (9*Z*)-α-carotene eluted before (all-*E*)-β-carotene.

(all-*E*)-Lycopene (peak 48; Rt = 43.1 min) was identified with *m*/*z* 537 and a characteristic λ_max_ at 443, 471, and 502 nm (%III/II = 6). MS/MS fragments were 457 [M + H − 80]^+^, 413 [M + H − 124]^+^, 177 [M + H − 360]^+^, 137 [M + H − 400]^+^, and 121 [M + H − 416]^+^. The presence of the carotene was confirmed with its authentic (all-*E*)-lycopene standard. Peaks 42, 44, 45 46, 47, and 49 were identified as *cis*-isomers of lycopene according to the MS/MS behavior, as well as with the reported literature [34]. According to the literature [33,35] related to carotenoid profile of papaya tissues, only the main hydrocarbon carotenoids (β-carotene and lycopene) are characterized. Nevertheless, Schweiggert et al. [34], in addition to β-carotene and lycopene, reported ζ-carotene and six lycopene isomers in yellow- and red-fleshed papaya. The carotenoid profile of Sweet Mary, Alicia, Eksotika, and Maradol papaya varieties showed five hydrocarbon carotenoids that have never been reported to be present in papaya fruits.

#### 3.2.3. Xanthophyll Esters in Papaya Fruits

Xanthophyll esters were mostly found in direct (non-saponified) pulp and peel extracts compared to saponified extracts (minor amounts of remaining xanthophyll esters after saponification process), in Sweet Mary variety (Figure 1) and within the other three studied papaya varieties (Figure S1, cv. Alicia, Figure S2, cv. Eksotika, and Figure S3, cv. Maradol, Supplementary Materials). Some of the most abundant xanthophyll esters were (all-*E*)-lutein-3-*O*-myristate (peak 28; Rt = 25.9; λ_max_ at 401, 426, and 472 nm), which was identified by comparing its UV/Vis spectrum and retention time with the literature reported by Rodrigues et al. [36], (all-*E*)-β-cryptoxanthin caprate (peak 35; Rt = 29.2; λ_max_ at 428, 450, and 476 nm), and (all-*E*)-β-cryptoxanthin laurate (peak 38; Rt = 30.3; λ_max_ at 421, 451, and 478 nm). (all-*E*)-Antheraxanthin myristate palmitate (peak 32; Rt = 27.4; λ_max_ at 421, 443, and 467 nm) was also found to be in high concentrations in direct papaya peel extracts.

Among other less abundant xanthophyll esters found in papaya pulp and peel extracts, we highlight (9*Z*)-violaxanthin laurate (peak 27; Rt = 25.6 min), (all-E)-violaxanthin dimyristate (peak 31; Rt = 27.2 min), and (9*Z*)-neoxanthin dibutyrate (peak 34; Rt = 29.0 min). The identification of (all-*E*)-β-cryptoxanthin myristate (peak 41; Rt = 32.1 min) and (all-*E*)-β-cryptoxanthin palmitate (peak 43; Rt = 33.9 min) was confirmed with the chromatographic characteristics described in Table 2 and with the reported literature [34].

In most of the papaya carotenoid characterization studies, a considerable number of free xanthophylls, hydrocarbon carotenoids, and xanthophyll esters are not reported. Schweiggert et al. [34] identified a total of 19 carotenoids of Costa Rican papaya hybrids and lines, of which only four carotenoids were identified as xanthophyll esters (β-cryptoxanthin caprate, β-cryptoxanthin laurate, β-cryptoxanthin myristate, and β-cryptoxanthin palmitate). The present study reports the identification of 11 more xanthophyll esters in Sweet Mary, Alicia, and Eksotika papaya varieties, as well as in cv. Maradol.

### 3.3. Carotenoid and Carotenoid Ester Content in Papaya Fruits

Carotenoid quantification of the three papaya varieties from the Canary Islands (Sweet Mary, Alicia, and Eksotika) in pulp and peel tissues is presented in Table 3 and Table 4, respectively. Furthermore, in these tables can be seen the carotenoid and carotenoid ester quantification in direct (non-saponified) and saponified extracts in pulp and peel fruit tissues. Furthermore, the same data for cv. Maradol are shown for comparison. The total carotenoid content in papaya pulps ranged from 1594.7 to 2147.5 μg/100 g fresh weight in direct extracts, and from 2930.4 to 3434.7 μg/100 g fresh weight in papaya peels. Sweet Mary, Alicia, and Eksotika varieties represent very high carotenoid sources (>2 mg/100 g fresh weight) according to the ranking proposed by Britton and Khachik [37]. In comparison with the carotenoid content of most popular Maradol papaya fruits, some authors [7] performed carotenoid analysis in papaya (*Carica papaya* L. cv Maradol) pulp in four stages of ripeness using HPLC-APCI-MS analysis. They found the highest carotenoid content (3.27 mg/100 g fresh weight) in the pulp with the most mature state (RS4), which fits with the mature state of the papayas used in this study (1.7 ± 49 mg/100 g fresh weight in Sweet Mary, 1.6 ± 40 mg/100 g fresh weight in Alicia, 2.2 ± 64 mg/100 g fresh weight in Eksotika, and 3.9 ± 84 mg/100 g fresh weight in Maradol variety). Additionally, Rivera-Pastrana et al. [35] studied the carotenoid content in papaya Maradol pulp during 12 days of storage at 1 °C and 90% relative humidity (RH) and at 25 °C at 60% RH. However, only a characterization of carotenoids compounds from saponified extracts was addressed. In the present study, we also completed the carotenoid profile in papaya Maradol tissues including the quantification of xanthophyll esters.

Total free xanthophyll content in Sweet Mary, Alicia, and Eksotika was 109.0, 84.6, and 120.5 μg/100 g fresh weight in pulp direct samples, while, in pulp saponified extracts, this content was 1204.5, 1257.0, and 1417.6 μg/100 g fresh weight (Table 3). In the Maradol variety, this content was slightly higher, showing 879.0 and 1566.5 μg/100 g fresh weight in direct and saponified extracts, respectively. The increase in free xanthophylls in saponified extracts is related to the removal of mono and di-fatty acids from the xanthophyll ester molecules present in the non-saponified extracts (Table 3 and Table 4). These data are also shown in Figures S3 and S4 (Supplementary Materials) to better represent the distribution of carotenoid composition of each papaya variety and tissue. Despite descriptive analyses of xanthophyll esters in papaya Maradol pulp or peel not being found in the literature, we include this information in the present study due to their biological importance, such as lutein esters. The study of lutein in either its free form or its esterified form may be interesting due to its beneficial effects on human health, since lutein may decrease the risk of developing age-related macular degeneration (AMD) and other eye diseases, while it may also be associated with cognitive function in adults [38]. For this reason, the use of papaya byproducts, such as peels, is a great way to extract high concentrations of this xanthophyll to obtain healthy ingredients.

The most abundant xanthophyll esters found in papaya pulps (Table 3) of the studied varieties (direct extract) followed the order (all-*E*)-β-cryptoxanthin laurate (174.9, 167.8, 223.2, and 281.7 μg/100 g fresh weight in Sweet Mary, Alicia, Eksotika, and Maradol) ≥ (all-*E*)-lutein-3-*O*-myristate (168.7, 210.7, 261.6, and 278.0 μg/100 g fresh weight in Sweet Mary, Alicia, Eksotika, and Maradol) ≥ (all-*E*)-β-cryptoxanthin caprate (81.8, 69.7, 90.4, and 91.6 μg/100 g fresh weight in Sweet Mary, Alicia, Eksotika, and Maradol) ≥ (all-*E*)-lutein dimyristate (60.3, 61.6, 79.7, and 92.1 μg/100 g fresh weight in Sweet Mary, Alicia, Eksotika, and Maradol). Schweiggert et al. [39] also found β-cryptoxanthin laurate (481 μg/100 g fresh weight) as one of the main xanthophyll esters in papaya Pococí (or Sweet Mary) variety, followed by β-cryptoxanthin caprate (219 μg/100 g fresh weight) and β-cryptoxanthin myristate (55 μg/100 g fresh weight).

In contrast, Table 4 shows that papaya peels had higher content of (all-*E*)-antheraxanthin myristate palmitate (146.3, 95.4, 125.6, and 70.2 μg/100 g fresh weight in Sweet Mary, Alicia, Eksotika, and Maradol) ≥ (all-*E*)-antheraxanthin-3-*O*-palmitate (74.7, 23.3, 120.2, and 54.8 μg/100 g fresh weight in Sweet Mary, Alicia, Eksotika, and Maradol) ≥ (all-*E*)-β-cryptoxanthin laurate (74.4, 66.8, 64.0, and 194.9 μg/100 g fresh weight in Sweet Mary, Alicia, Eksotika, and Maradol). To the best of our knowledge, no studies have published the identification and quantification of xanthophyll esters in papaya Maradol peel; however, they are included in this study for comparison with the results obtained from the analysis of the three new papaya varieties from the Canary Islands (Spain) and to provide information about new ways of using papaya peel, considered as a byproduct, in order to grant it added value by obtaining carotenoid extracts for healthy ingredients.

Among all the hydrocarbon carotenoids found in Sweet Mary, Alicia, and Eksotika, (all-*E*)-β-carotene and (all-*E*)-lycopene were the most abundant in pulps and peels, as also found in the Maradol variety. The Eksotika variety showed the highest content of (all-*E*)-β-carotene (170.3 μg/100 g fresh weight) and (all-*E*)-lycopene (421.2 μg/100 g fresh weight) in pulp. When comparing the content of hydrocarbon carotenoids in peels, cv. Sweet Mary showed the highest (all-*E*)-β-carotene content (233.2 μg/100 g fresh weight), and cv. Eksotika showed the highest content in (all-*E*)-lycopene (318.8 μg/100 g fresh weight).

Figures S5 and S6 (Supplementary Materials) shows the carotenoid proportion (%) of carotenoid families (free xanthophylls, hydrocarbon carotenoids and xanthophyll esters) and carotenoid species in direct extracts and in saponified extracts of the three papaya varieties from the Canary Islands (Spain) and of the Maradol papaya variety from Mexico. One of the most abundant carotenes found in the pulp of these varieties was (all-*E*)-lycopene, showing a range of 25–39% in direct extracts and 25–29% in saponified extracts. The observed differences in (all-*E*)-lycopene content in direct and saponified extracts were not statistically different (*p* ≤ 0.05), which is an expected result due to the low alteration of hydrocarbon carotenoids during the saponification process. During this process, some oxidation reactions of lutein esters seem to take place, producing slightly lower amounts of this free carotenoid that could be expected in saponified extract analysis (16–17% direct extracts; 6–12% saponified extracts). In papaya peels, (all-*E*)-violaxanthin and its isomers showed higher ranges (7–17% direct extracts; 14–19% saponified extracts) than the other carotenoid species. This xanthophyll (and its isomers) follows the xanthophyll cycle, where the violaxanthin pool can be de-epoxidized to zeaxanthin via the intermediate antheraxanthin, which showed a range of 6–9% in direct peel extracts and around 5% in saponified extracts. Shen et al. [40] related carotenoid biosynthesis pathways to color change and carotenoid content in papaya tissues. They reported the presence of β-cryptoxanthin, zeaxanthin, and lycopene, as well as of β-carotene and lutein, in papaya saponified peel extracts, which is related to the data obtained in the present investigation.

Schweiggert et al. [34] studied the qualitative composition of carotenoids of red- and yellow-fleshed papaya lines and hybrids. In their investigation, the levels of pro-vitamin A carotenoids presented by Pococí (or Sweet Mary) papaya variety (233 and 514 μg/100 g fresh of weight of β-cryptoxanthin and β-carotene content, respectively) were slightly higher compared to the content of β-cryptoxanthin (43.1 μg/100 g fresh of weight) and β-carotene (165 μg/100 g fresh weight) found in Sweet Mary variety of the present investigation (Table 3).

Pro-vitamin A potential was obtained calculating retinol activity equivalent (RAE) according to the United States (US) Institute of Medicine [27]. Eksotika and Sweet Mary varieties showed similar pro-vitamin A potential in their edible tissue (23.4 and 22.7 RAE) in comparison to Alicia fruits (18.0 RAE) (Table 3). According to Wall [41], papaya cv. Maradol presents an RAE of 45.6 μg RAE/100 g fresh weight, which is similar to that for papaya cv. Maradol reported in this investigation (41.1 μg RAE/100 g fresh weight). RAE was also calculated in papaya peels, obtaining the highest value in Sweet Mary and Eksotika peel extracts (36.7 μg RAE/100 g fresh weight), followed by Maradol and Alicia varieties (34.0 and 28.7 μg RAE/100 g fresh weight, respectively). Since papaya peel is generally discarded by consumers, papaya pulp becomes an edible good source of pro-vitamin A [33]. However, as it has been demonstrated, papaya peel could be an interesting source to obtain carotenoid-rich extracts with pro-vitamin A potential.

### 3.4. Carotenoid Deposition in Sweet Mary, Alicia, and Eksotika Papaya Fruits

**Figure 2:**
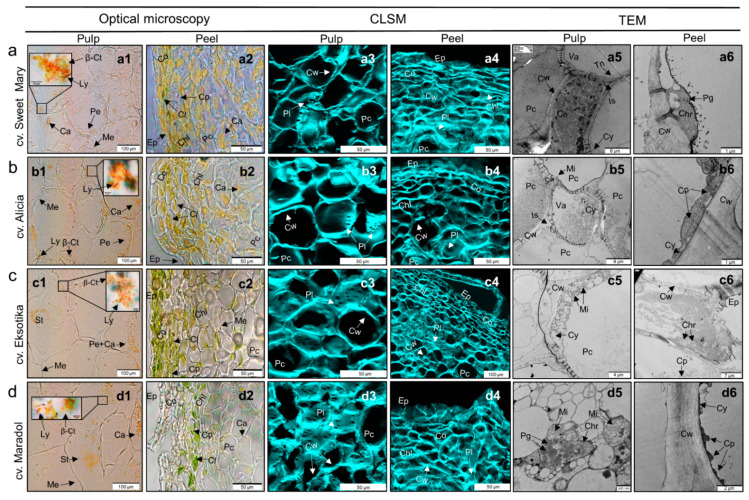
Optical, confocal laser scanning (CLSM), and transmission electron microscopy (TEM) images of (**a**) Sweet Mary, (**b**) Alicia, (**c**) Eksotika, and (**d**) Maradol papaya varieties. β-Ct: β-carotene globular chromoplasts, Ca: carotenoids, Chl: chlorenchyma cell, Chr: chromoplast, Ce: companion cell, Cy: cytoplasm, Cl: chlorophyll, Co: collenchyma cell, Cp: chloroplast, Cw: cell wall, Ep: upper epidermis, Is: intercellular space, Me: membrane, Mi: mitochondria, Ly: lycopene crystalloid formation, Pc: parenchyma cell, Pg: plastoglobuli, Pe: pectin, Pe + Ca: carotenoids surrounded by pectin, Pl: plasmodesma, St: starch granules, Tn: tonoplast, and Va: vacuole.

Papaya fruits, as with most fruits and vegetables, are composed of different sections: collenchyma, chlorenchyma, and parenchyma cells. The peel of the fruit is formed by collenchyma and chlorenchyma cells, while the pulp contains parenchyma cells, as shown in Figure 2. Carotenoid deposition in Sweet Mary, Alicia, Eksotika, and Maradol papaya pulps and peels, in the same maturity stage (stage 5), was analyzed using optical, confocal laser scanning, and transmission electron microscopy to study the potential carotenoid bioavailability.

Optical microscopy showed that parenchyma cells in the pulp of all papaya varieties were the largest cells in the tissue sections. These types of cells presented their characteristic turgidity, with small intercellular space and thin cell walls. The carotenoid accumulation (chromoplasts) in the three papaya varieties from the Canary Islands (Figure 2a1, b1, c1) and in the Mexican variety (Figure 2d1) was mainly localized around the edge of the parenchyma cells, close to the membrane, showing the presence of some crystalloid and round-shaped structures. The carotenoid location was due to the presence of large vacuoles (as demonstrated with TEM in Figure 2a5–d5) inside the cells, which displaced the organelles to the outer part of the cell. Several investigations related to the chromoplast microstructure of different fruits (lucuma, mango, tangerine tomato, etc.) were extensively performed during the last few years [17,42,43]. Schweiggert and Carle [18] mentioned that the deposition of carotenoid in fresh and processed foods is strongly related to carotenoid bioavailability due to micelle formation and absorption in the small intestine. According to these studies, chromoplasts of papaya fruits present a tubular (or globular–tubular) shape, containing mostly liquid-crystalline carotenoids in the core of tubules. This fact was confirmed by Schweiggert et al. [44], revealing that the in vitro bioaccesibility of β-carotene from mango and papaya (tubular shape) was significantly higher than that from carrots and tomatoes (globular chromoplasts). Furthermore, chromoplasts in red-fleshed papaya fruits (such as Sweet Mary, Eksotika, and Alicia varieties) may be found as crystalloid chromoplasts, where the carotenoid (commonly β-carotene and/or lycopene) forms crystals [40]. Sweet Mary, Eksotika, and Maradol light micrographs (Figure 2a1, c1, and d1) exhibited mostly elongated crystalloids, compared to globular depositions, contrary to the Alicia variety (Figure 2b1), which presented more globular forms. These results are correlated with Schweiggert et al.’s [39] investigation, where they also reported these carotenoid structures in red- and yellow-fleshed papaya (*Carica papaya* L. cv. Sinta and Pococí, respectively). They also reported that the accumulation of (all-*E*)-lycopene in lycopene-rich fruits forms sharp crystalloids due to its poor solubility and its continuous accumulation in the tissues. Contrarily, lycopene *Z*-isomers and, generally, β-carotene are gathered into globular structures, mainly during the ripening process, being more characteristic during unripened stages. Thus, the differences between crystalloid sizes and distribution is directly associated with different concentrations among (all-*E*)-lycopene and lycopene *Z*-isomers presented by these papaya varieties. The presence of different carotenoids in the chromoplasts, such as lutein and its esters, has been proven to play an important role in the bioavailability of bioactive compounds. Despite all the mentioned benefits that lutein may have for human health, several studies reported its low bioavailability [38,45]. One of the most plausible solutions would be diet supplementation with lutein, extracted from papaya peels due to its higher concentration in this tissue than in papaya pulps, as a healthy ingredient to develop antioxidant formulas using microencapsulation techniques, due to its limited solubility in grade solvents (oils) and its insolubility in aqueous systems [46]. On the other hand, peel light micrographs (Figure 2a2–d2) clearly showed abundant pectin and hemicellulose accumulations in the epidermis and directly under the collenchyma cells, due to their supporting function. Chlorenchyma cells were distinguished because of the presence of numerous chloroplasts within the cells, which were more abundant in cv. Eksotika compared to the other papaya varieties. Carotenoids were also observed in all papaya peel varieties, with Sweet Mary being the variety with the highest carotenoid accumulation. No crystalloids or globular depositions were found in any of the peel sections. However, the presence of pectin substances around the carotenoids was remarkable in both pulp and peel of the different papaya varieties.

Confocal laser scanning microscopy (CLSM) showed that pulp cell walls in the Canary Islands papaya varieties (Figure 2a3–c3) had similar shape and size, but Maradol pulp cells (Figure 2d3) were revealed to be smaller in size and less rounded. Moreover, CLSM allowed distinguishing cellular structures such as microstructural channels, named plasmodesma, which allow transversal communication between vegetable cells. Although, no remarkable differences were found with CLSM between peels of the different papaya varieties (Figure 2a4–d4), there were noticeably thicker cell walls in peel cells than in pulp cells, as well as a higher number of plasmodesma in the cells of the pulp than in peel cells.

Cell structures and organelles were further analyzed by transmission electron microscopy (TEM). As observed with optical microscopy, large vacuoles were identified in most of the pulp cells of Sweet Mary, Alicia, Eksotika, and Maradol varieties (Figure 2a5–d5). In the reduced cytoplasm sections, mitochondria were observed, as well as chromoplasts (Figure 2d5). These structures were distinguished due to the smaller dimensions and cristae characteristic of mitochondria. Inside chromoplasts, numerous plastoglobules (electron-dense structures) were identified. Schweiggert et al. [39] were also able to distinguish these organelles in papayas from Costa Rica. On the other hand, it was also possible to identify chromoplasts containing plastoglobules in papaya peel sections (Figure 2a6), as well as chloroplasts, which are characteristic of this part of the fruit. They were observed close to the edge of the vegetable cell and identified by their characteristic double membrane and grana structures (Figure 2b6–d6). No differences among varieties were found.

Further studies should be carried out to determine if carotenoid aggregation within the vegetable cell may influence carotenoid absorption in the gastrointestinal tract and, therefore, bioavailability. According to a recent study conducted by Cano et al. [15], the modification of the composition and characteristics of polysaccharides (mainly pectin) in persimmon tissues could be responsible for increasing carotenoid bioaccessibility in this fruit. High pectin content in the food matrix hinders micelle formation, leading to low carotenoid bioaccessibility. As pectin substances are part of the fiber, carotenoid bioaccessibility could also be hampered by high fiber content in some fruits and vegetables [18,47,48]. This work will be continued with further investigations to gather more information about carotenoid stability and bioavailability to define those carotenoids and carotenoid esters which are potentially absorbable after the consumption of the edible parts of fruits and vegetables.

## 4. Conclusions

This paper describes, for the first time, the complete carotenoid and carotenoid ester profile and the carotenoid deposition in plastids of fruits of three new varieties of papaya (*Carica papaya* L.), Sweet Mary, Alicia, and Eksotika, from the Canary Islands (Spain) at full maturity. Additionally, a comparison was made with the carotenoid and carotenoid ester composition of fruits of papaya cv. Maradol. The carotenoid profile of the peel and pulp of these new papaya varieties revealed a total of 46 carotenoid compounds corresponding to 16 free xanthophylls, 15 hydrocarbon carotenoids, and 15 xanthophyll esters. The most abundant xanthophyll esters found in papaya pulps were (all-*E*)-β-cryptoxanthin laurate, (all-*E*)-lutein-3-*O*-myristate, and (all-*E*)-lutein dimyristate. In contrast, papaya peels had a higher content of (all-*E*)-antheraxanthin myristate palmitate, (all-*E*)-antheraxanthin-3-*O*-palmitate, and (all-*E*)-β- cryptoxanthin laurate. With respect to free xanthophylls, the most abundant in direct extracts (non-saponified) of papaya pulps were (all-*E*)-β-cryptoxanthin and (all-*E*)-antheraxanthin, whereas (all-*E*)-lutein was only found in Maradol papaya pulp direct extract. In terms of the papaya peels, the most abundant free xanthophylls in direct extracts from peel tissue were (all-*E*)-lutein in all papaya varieties. (all-*E*)-Lycopene and (all-*E*)-β-carotene were the most abundant hydrocarbon carotenoids in peel and pulp tissues in direct extracts of all studied varieties of papaya fruits.

Carotenoids in papaya pulp tissues were deposited in tubular chromoplasts in the periphery of the cells. Lycopene and β-carotene crystalloids were observed due to their low solublility in the cell matrix. No crystalloids or globular depositions were found in any of the papaya peel sections. However, the presence of pectin substances around the carotenoids was remarkable in both pulp and peel of the different studied papaya varieties. The observed differences in carotenoid deposition found by comparing pulp cells of the four papaya varieties could be related to the interactions of the carotenoids and enzymes during gastrointestinal digestion, which may affect the stability and bioaccessibility of carotenoids. In this sense, a further study with these Canarian papaya varieties will be conducted to gain insight into the behavior of their carotenoids using in vitro and in vivo gastrointestinal digestion models and to evaluate the influence of papaya macroconstituents such as polysaccharides (mainly pectins and cellulose) and microestructure on carotenoid bioaccessibity and stability.

## Figures and Tables

**Table 1:** Physical and physicochemical characteristics of papaya (*Carica papaya* L.) Sweet Mary, Alicia, and Eksotika varieties from the Canary Islands (Spain) and cv. Maradol from Mexico.

Characteristic	cv. Sweet Mary	cv. Alicia	cv. Eksotika	cv. Maradol
Fruit appearance	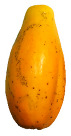	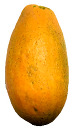	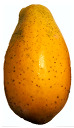	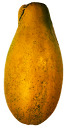
Total weight of whole fruit (g)	1124.0 ± 214.2 ^a^	1286.1 ± 155.3 ^a^	1148.2 ± 204.3 ^a^	1350.0 ± 251.1 ^b^
Apical caliber (cm)	19.1 ± 5.2 ^a^	20.5 ± 0.8 ^a^	19.4 ± 2.0 ^a^	26.5 ± 3.8 ^b^
Equatorial caliber (cm)	10.7 ± 0.7 ^a^	11.6 ± 0.3 ^a^	11.5 ± 0.4 ^a^	10.9 ± 0.3 ^a^
Titratable acidity ^1^	0.184 ± 0.000 ^a^	0.182 ± 0.005 ^a^	0.190 ± 0.002 ^a^	0.179 ± 0.02 ^b^
pH	5.275 ± 0.007 ^a^	5.350 ± 0.028 ^b^	5.430 ± 0.000 ^c^	5.487 ± 0.019 ^c^
Soluble solids (°Brix at 25 ⁰C)	12.2 ± 0.2 ^b^	11.2 ± 0.1 ^a^	12.1 ± 0.2 ^b^	12.5 ± 0.1 ^b^
Moisture content (% wet basis)	83.4 ± 0.2 ^a^	84.6 ± 0.3 ^b^	85.1 ± 0.2 ^c^	83.7 ± 0.1 ^a^
Pulp color parameters				
L*	63.1 ± 1.1 ^a^	62.3 ± 0.9 ^a^	63.0 ± 3.7 ^a^	62.7 ± 1.6 ^a^
a*	28.1 ± 1.2 ^c^	23.9 ± 0.3 ^b^	19.8 ± 1.0 ^a^	28.1 ± 1.7 ^c^
b*	42.8 ± 2.1 ^b^	40.4 ± 1.1 ^b^	30.8 ± 0.8 ^a^	42.8 ± 2.1 ^b^
Hue angle (h*)	56.6 ± 0.3 ^b^	59.3 ± 1.0 ^c^	57.3 ± 0.7 ^b^	49.4 ± 0.7 ^a^
Peel color parameters				
L*	56.0 ± 0.7 ^a^	50.3 ± 1.1 ^a^	59.0 ± 2.2 ^a^	56.0 ± 0.7 ^a^
a*	13.7 ± 0.9 ^a^	15.2 ± 0.5 ^a^	14.1 ± 0.5 ^a^	17.7 ± 0.4 ^a^
b*	24.4 ± 0.6 ^a^	22.9 ± 1.4 ^a^	26.4 ± 3.6 ^a^	24.4 ± 0.6 ^a^
hue angle (h*)	60.8 ± 1.1 ^b^	56.4 ± 1.3 ^a^	61.7 ± 2.5 ^b^	54.1 ± 0.9 ^a^

Values are the mean of three independent determinations ± standard deviation. Different superscript letters within a row indicate statistically significant differences (*p* ≤ 0.05). ^1^ g citric acid/100 g fresh weight. L*, lightness; a*, green–red tonality; b*, blue–yellow tonality.

**Table 2:** HPLC retention times, ultraviolet (UV)/visible light (Vis) spectra, and MS spectral data of carotenoids from papaya (*Carica papaya* L.) pulp and peel of Sweet Mary, Alicia, Eksotika, and Maradol varieties.

No.	Rt (min)	Compound Identity	HPLC-DAD UV/Vis Absorption Maxima (nm)	%III/II	%A_b_/A_II_	[M + H]^+^ *m/z*	HPLC/APCI^+^ MS Fragmentation Pattern *(m/z)* Fragment ions *(m/z)*	STD ^c^
1	3.4	(13*Z*)-violaxanthin ^a^	326, 416, 440, 470	78	0	601	583 [M + H − 18]^+^, 565 [M + H − 36]^+^, 509 [M + H − 92]^+^, 491 [M + H − 92 − 18]^+^,	n.a.
2	3.8	(all-*E*)-violaxanthin	414, 438, 468	98	0	601	583 [M + H − 18]^+^, 565 [M + H − 36]^+^, 521 [M + H − 80]^+^	Y
3	4.2	not identified 1	400, 422, 448	0	0	nd ^g^	nd ^g^	n.a.
4	5.2	(9Z)-neoxanthin	327, 410, 432, 462	55	0	601	583 [M + H − 18]^+^, 565 [M + H − 36]^+^, 547 [M + H − 54]^+^, 521 [M + H − 80]^+^	n.a.
5	7.6	(all-*E*)-neoxanthin	416, 437, 469	99	0	601	583 [M + H − 18]^+^, 565 [M + H − 36]^+^, 547 [M + H − 54]^+^, 521 [M + H − 80]^+^	Y
6	7.9	(all-*E*)-lutein	(420), 444, 472	62	0	569	551 [M + H − 18]^+^, 533 [M + H − 36]^+^	Y
7	9.5	(all-*E*)-zeaxanthin	(426), 450, 470	18	0	569	551 [M + H − 18]^+^, 533 [M + H − 36]^+^	Y
8	10.3	lutein-5,6-epoxide	(418), 440, 468	85	nc ^f^	585	567 [M + H − 18]^+^, 549 [M + H − 36]^+^, 505 [M + H − 80]^+^	n.a.
9	10.9	(all-*E*)-antheraxanthin	(422), 444, 472	66	0	585	567 [M + H − 18]^+^, 549 [M + H − 36]^+^, 505 [M + H − 80]^+^	n.a.
10	12.3	(9Z)-violaxanthin	327, 414, 436, 468	89	nc ^f^	601	583 [M + H − 18]^+^, 565 [M + H − 36]^+^	n.a.
11	13.2	(all-*E*)-β-apo-caroten-8’-al (internal standard)	462	0	0	417	399 [M + H − 18]^+^, 325 [M + H − 74]^+^	Y
12	13.7	β-cryptoxanthin-5,6-epoxide	(420), 445, 471	52	nc ^f^	569	551 [M + H − 18]^+^, 459 [M + H − 18 − 92]^+^, 221	n.a.
13	14.4	(9Z)-α-cryptoxanthin ^d^	412, 437, 466	0	0	nd ^g^	nd ^g^	n.a.
14	14.7	not identified 2	437, 457, 488	0	0	nd ^g^	nd ^g^	n.a.
15	17.2	(all-*E*)-α-cryptoxanthin ^b^	(413), 435, 464	61	nc ^f^	553	535 [M + H − 18]^+^, 479, 461 [M + H − 92]^+^, 439	Y
16	18.1	(all-*E*)-β-cryptoxanthin	(426), 450, 476	18	nc ^f^	553	535 [M + H − H_2_O]^+^, 461 [M + H − 92]^+^	Y
17	19.3	α-carotene-5,6-epoxy-	(418), 441, 469	10	0	553	535 [M + H − 18]^+^, 495, 205	n.a.
18	19.7	(all-*E*)-luteoxanthin	400, 416, 444	nc ^f^	0	601	583 [M + H − 18]^+^	n.a.
19	20.3	(13*Z*)-α-carotene	337, (413), 439, 467	31	0	537	481 [M + H − 56]^+^, 445 [M + H − 92]^+^	n.a.
20	21.2	(13*Z*)-β-carotene ^c^	337, (414), 436, 464	14	0	537	457 [M + H − 80]^+^, 445 [M + H − 92]^+^, 400 [M + H − 137]^+^, 269 [M + H − 268]^+^, 177 [M + H − 360]^+^, 137 [M + H − 400]^+^	n.a.
21	21.8	(all-*E*)-violaxanthin laurate ^d^	417, 441, 469	nc ^f^	0	783	765 [M + H − 18]^+^, 747 [M + H − 18 − 18]^+^, 691 [M + H − 92]^+^, 673 [M + H − 92 − 18]^+^, 583 [M + H − 12:0]^+^, 565 [M + H − 12:0 − 18]^+^, 547 [M + H − 12:0 − 18 − 18]^+^	n.a.
22	22.1	β-cryptoxanthin-5,8-epoxide	412, 438, 464	50	0	569	551 [M + H − 18]^+^, 459 [M + H − 18 − 92]^+^,221	n.a.
23	22.5	(all-*E*)-ζ-carotene	378, 400, 423	108	0	541	472 [M + H − 69]^+^, 404 [M + H − 137]^+^,364,337	n.a.
24	23.4	β-cryptoxanthin-5,8’-epoxide	413, 438, 465	50	0	569	nd ^g^	n.a.
25	24.0	(all-*E*)-α-carotene	(420), 445, 470	66	0	537	457 [M + H − 80]^+^, 413 [M + H − 124]^+^, 177 [M + H − 360]^+^, 137 [M + H − 400]^+^, 123 [M + H − 414]^+^	Y
26	24.7	(9Z)-α-carotene	396, 420, 440, 469	60	0	537	457 [M + H − 80]^+^, 445 [M + H − 92]^+^	n.a.
27	25.6	(9Z)-violaxanthin laurate	388, 413, 436, 466	92	0	783	765 [M + H − H_2_O]^+^, [M + H − 18]^+^,747 [M + H − 2H_2_O]^+^, [M + H − 18 − H_2_O]^+^, 565 [M + H − 12:0 − H_2_O]^+^, [M + H − 12:0 − 18]^+^	n.a.
28	25.9	(all-*E*)-lutein-3-*O*-myristate	401, 426, 472	0	0	nd ^g^	533 [M + H − 228 − 18]^+^, 495 [M + H − 228 − 56]^+^, 459 [M + H − 228 − 92]^+^, 429, 441	n.a.
29	26.2	(all-*E*)-β-carotene ^c^	(428), 450, 476	16	0	537	457 [M + H − 80]^+^, 445 [M + H − 92]^+^, 400 [M + H − 137]^+^, 269 [M + H − 268]^+^, 177 [M + H − 360]^+^, 137 [M + H − 400]^+^	Y
30	26.8	(9Z)-β-carotene	380, 400, 426, 454	23	0	537	457 [M + H − 80]^+^, 445 [M + H − 92]^+^, 400 [M + H − 137]^+^, 269 [M + H − 268]^+^, 177 [M + H − 360]^+^, 137 [M + H − 400]^+^	n.a.
31	27.2	(all-*E*)-violaxanthin dimyristate	413, 435, 464	91	0	10220	1004 [M + H − H_2_O]^+^, 793 [M + H − 14:0]^+^, 775 [M + H − 14:0 − H_2_O]^+^, 547 [M + H − 14:0 − 14:0 − H_2_O]^+^	n.a.
32	27.4	(all-*E*)-antheraxanthin myristate palmitate	421, 443, 467	31	0	1033	1015 [M + H − 18]^+^, 941 [M + H − 92]^+^, 805 [M + H − 14:0]^+^, 787 [M + H − 14:0 − 18]^+^, 771 [M + H − 16:0]^+^,759 [M + H − 16:0 − 18]^+^, 549 [M + H − 14:0 − 16:0]^+^, 531 [M + H − 14:0 − 16:0 − 18:0]^+^	n.a.
33	27.9	(all-*E*)-violaxanthin palmitate ^d^	416, 441, 469	nc ^f^	nc ^f^	839	821 [M + H − 18]^+^, 803 [M + H − 18 − 18]^+^, 747 [M + H − 16:0]^+^, 729 [M + H − 92 − 18]^+^, 583 [M + H − 256]^+^, 565 [M + H − 18 − 16:0]^+^, 547 [M + H − 16:0 − 18 − 18]^+^	n.a.
34	29.0	(9Z)-neoxanthin dibutyrate ^d^	327, 412, 436, 464	80	16	741	723 [M + H − 18]^+^, 653 [M + H − 4:0]^+^, 649 [M + H − 92]^+^, 635 [M + H − 4:0 − 18]^+^, 631 [M + H − 92 − 18]^+^, 565 [M + H − 4:0 − 4:0]^+^, 547 [M + H − 4:0 − 4:0 − 18]^+^	n.a.
35	29.2	(all-*E*)-β-cryptoxanthin caprate ^d^	428, 450, 476	nc ^f^	nc ^f^	707	615 [M + H − 27]^+^, 535 [M + H − 100]^+^, 443 [M + H − 11]^+^, 442 [M + H − 16]^+^	n.a.
36	29.8	(9Z)-violaxanthin myristate palmitate	413, 439, 467	92	0	1050	1032 [M + H − H_2_O]^+^, 803 [M + H − 14:0 − H_2_O]^+^, 775 [M + H − 16:0 − H_2_O]^+^, 565 [M + H − 14:0 − 16:0]^+^, 547 [M+14:0 − 16:0 − H_2_O]^+^,	n.a.
37	30.0	(all-*E*)-lutein dimyristate	422, 446, 474	38	0	nd ^g^	761 M + H − 14:0]^+^, 669 [M + H − 92]^+^, 553 [M + H − 14:14:0]^+^	n.a.
38	30.3	(all-*E*)-β-cryptoxanthin laurate	421, 451, 478	25	0	735	643 [M + H − 92]^+^, 535 [M + H − 12:0]^+^, 479 [M + H − 56 − 12:0]^+^, 443 [M + H − 92 − 12:0]^+^	
39	30.4	(all-*E*)-antheraxanthin-3-*O* palmitate	422, 444, 472	nc ^f^	nc ^f^	823	805 [M + H − 18]^+^, 787 [M + H − 18 − 18]^+^, 731 [M + H − 92]^+^, 567 [M + H − 16:0]^+^, 549 [M + H − 16:0 − 18]^+^, 531 [M + H − 16:0 − 18 − 18]^+^	n.a.
40	31.7	(all-*E*)-antheraxanthin laurate myristate ^d^	418, 442, 470	33	0	977	959 [M + H − 18]^+^, 777 [M + H − 12:0]^+^, 749 [M + H − 14:0]^+^, 759 [M + H − 12:0 − 18]^+^, 731 [M + H − 14:0 − 18]^+^, 549 [M + H − 12:0 − 14:0]^+^, 531 [M + H − 12:0 − 14:0 − 18]^+^	n.a.
41	32.1	(all-*E*)-β-cryptoxanthin myristate	424, 448, 476	9	0	763	671 [M + H − 92]^+^, 535 [M + H − 14:0]^+^, 443 [M + H − 14:0 − 92]^+^	n.a.
42	33.3	(Z)-lycopene isomer 1 ^d^	417, 442, 469, 499	0	0	537	481 [M + H − 51]^+^, 467 [M + H − 49]^+^, 455 [M + H − 100]^+^, 427 [M + H − 15]^+^, 413 [M + H − 43]^+^, 399 [M + H − 64]^+^, 387 [M + H − 11]^+^	n.a.
43	33.9	(all-*E*)-β-cryptoxanthin palmitate ^d^	433, 460, 487	0	0	791	699 [M + H − 39]^+^, 535 [M + H − 100]^+^, 443 [M + H − 4]^+^, 413 [M + H − 46]^+^	n.a.
44	34.0	(13Z)-lycopene isomer 2 ^d^	442, 465, 493	0	0	537	481 [M + H − 42]^+^, 467 [M + H − 35]^+^, 455 [M + H − 100]^+^, 427 [M + H − 61]^+^, 413 [M + H − 88]^+^, 399 [M + H − 24]^+^, 387 [M + H − 42]^+^	n.a.
45	34.9	(13’Z)-lycopene isomer 3 ^d^	437, 460, 490	0	0	537	481 [M + H − 5]^+^, 467 [M + H − 42]^+^, 455 [M + H − 86]^+^, 427 [M + H − 22]^+^, 413 [M + H − 4]^+^, 399 [M + H − 86]^+^, 387 [M + H − 11]^+^	n.a.
46	38.4	(9Z)-lycopene isomer 4 ^d^	440, 465, 496	0	0	537	481 [M + H − 11]^+^, 467 [M + H − 32]^+^, 455 [M + H − 79]^+^, 427 [M + H − 48]^+^, 413 [M + H − 30]^+^, 399 [M + H − 42]^+^, 387 [M + H − 38]^+^	n.a.
47	38.9	(9’Z)-lycopene isomer 5 ^d^	413, 439, 465, 496	0	0	537	481 [M + H − 49]^+^, 467 [M + H − 20]^+^, 455 [M + H − 100]^+^, 427 [M + H − 24]^+^, 413 [M + H − 60]^+^, 399 [M + H − 21]^+^, 387 [M + H − 19]^+^,	n.a.
48	43.1	(all-*E*)-lycopene	418, 443, 471, 502	6	0	537	457 [M + H − 80]^+^, 413 [M + H − 124]^+^, 177 [M + H − 360]^+^, 137 [M + H − 400]^+^, 121 [M + H − 416]^+^	Y
49	43.4	(Z)-lycopene isomer 6 ^d^	443, 471, 502	0	0	537	481 [M + H − 17]^+^, 467 [M + H − 32]^+^, 455 [M + H − 100]^+^, 427 [M + H − 23]^+^, 413 [M + H − 61]^+^, 399 [M + H − 40]^+^, 387 [M + H − 17]^+^	n.a.

DAD, diode array detection; APCI^+^, atmospheric pressure chemical ionization with positive ion mode; Rt, retention time; n.d., not detected; n.a., not available. ^a^ Tentatively identified by comparing the DB/DII value of the putative (9Z)-violaxanthin (0.06) to that reported by Molnár et al. [29], i.e., 0.06 (Q = 17.50). ^b^ In source fragment [M +H − H_2_O]^+^ in agreement with reference compound. ^c^ Tentatively identified by comparing the DB/DII values of the putative (9Z)-β-carotene (0.06) and (13Z)-β-carotene (0.42) to those reported by Meléndez-Martínez et al. [30], i.e., 0.13 and 0.43, respectively. ^d^ Tentative identification according to UV/Vis spectrum. The obtained mass spectra were highly ambiguous, hindering its reliable identification. “Y” indicates that the presented analytical data were in agreement with an authentic reference compound. ^f^ %III/II was not calculated because of the poor definition of the UV/Vis spectrum or because it was not detected. ^g^ [M + H]^+^ or MS/MS fragments were not detected. Fatty acid refers to the following mass losses: 88 u = (4:0) butyric acid; 200 u = (12:0) lauric acid; 228 u = (14:0) myristic acid; 256 u = (16:0) palmitic acid; 284 u = (18:0) stearic acid.

**Table 3:** Carotenoid content ^1^ (μg/100 g fresh weight) (mean ± standard deviation) and retinol activity equivalent (RAE) of direct and saponified extracts from papaya (*Carica papaya* L.) pulps of Sweet Mary, Alicia, Eksotika, and Maradol varieties.

No.^2^	Carotenoid Compound	cv. Sweet Mary		cv. Alicia		cv. Eksotika		cv. Maradol	
		Direct Extract (C)	Saponified Extract (SAP)	Direct Extract (C)	Saponified Extract (SAP)	Direct Extract (C)	Saponified Extract (SAP)	Direct Extract (C)	Saponified extract (SAP)
1	(13*Z*)-violaxanthin	1.2 ± 0.1 ^a^	112.7 ± 9.4 ^c^	n.d ^a^	11.2 ± 0.1 ^a^	n.d ^a^	124.4 ± 7.7 ^b^	37.1 ± 1.8 ^b^	136.5 ± 0.6 ^c^
2	(all-*E*)-violaxanthin	3.4 ± 0.1 ^b^	115.3 ± 7.8 ^c^	n.d ^a^	114.3 ± 3.8 ^a^	n.d ^a^	151.6 ± 3.9 ^b^	52.8 ± 2.1 ^c^	147.6 ± 0.2 ^bc^
3	not identified 1	tr.	tr.	tr.	tr.	tr.	tr.	tr.	tr.
4	(9*Z*)-neoxanthin	n.d ^a^	176.8 ± 2.0 ^c^	n.d ^a^	166.2 ± 3.6 ^a^	n.d ^a^	151.1 ± 2.1 ^a^	5.8 ± 0.1 ^b^	150.4 ± 2.2 ^b^
5	(all-*E*)-neoxanthin	n.d ^a^	37.5 ± 2.9 ^b^	n.d ^a^	n.d ^a^	n.d ^a^	n.d ^a^	30.7 ± 1.6 ^b^	91.4 ^c^
6	(all-*E*)-lutein	n.d ^a^	72.8 ± 0.9 ^a^	n.d ^a^	108.1 ± 2.7 ^c^	n.d ^a^	116.6 ± 9.7 ^b^	142.2 ± 3.5 ^b^	162.1 ± 1.4 ^a^
7	(all-*E*)-zeaxanthin	n.d ^a^	61.4 ± 1.1 ^a^	15.0 ± 1.0 ^b^	95.5 ± 1.8 ^c^	27.5 ± 1.8 ^c^	67.4 ± 4.7 ^b^	88.5 ± 0.6 ^d^	196.8 ± 3.6 ^c^
8	lutein-5,6-epoxide	n.d ^a^	47.8 ± 1.3 ^a^	n.d ^a^	81.4 ± 0.3 ^b^	n.d ^a^	63.5 ± 0.1 ^a^	62.5 ± 4.8 ^b^	190.2 ± 2.5 ^c^
9	(all-*E*)-antheraxanthin	14.5 ± 0.8 ^b^	44.4 ± 3.6 ^b^	n.d ^a^	118.5 ± 2.3 ^b^	n.d ^a^	60.4 ± 1.0 ^a^	49.4 ± 1.7 ^c^	123.8 ± 2.2 ^c^
10	(9Z)-violaxanthin	6.0 ± 0.2 ^c^	29.0 ± 1.4 ^b^	n.d ^a^	33.9 ± 0.7 ^d^	n.d ^a^	5.7 ± 0.4 ^c^	20.7 ± 0.4 ^b^	n.d ^a^
11	(all-*E*)-β-apo-caroten-8’ al (IS^3^)	147.0 ± 0.6 ^a^	133.4 ± 0.5 ^a^	129.5 ± 0.3 ^a^	133.4 ± 0.5 ^a^	135.2 ± 0.3 ^a^	135.8 ± 0.6 ^a^	147.0 ± 0.6 ^a^	147.0 ± 0.6 ^a^
12	β-cryptoxanthin-5, 6-epoxide	n.d ^a^	46.8 ± 0.5 ^ab^	n.d ^a^	59.1 ± 0.4 ^a^	n.d ^a^	69.0 ± 3.2 ^c^	19.4 ± 1.2 ^b^	74.4 ± 0.3 ^bc^
13	(9*Z*)-α-cryptoxanthin	n.d ^a^	10.8 ± 0.4 ^c^	n.d ^a^	n.d ^a^	n.d ^a^	n.d ^a^	13.3 ± 0.3 ^b^	9.9 ± 0.2 ^b^
14	not identified 2	tr.	tr.	tr.	tr.	tr.	tr.	tr.	tr.
15	(all-*E*)-α-cryptoxanthin	2.5 ± 0.1 ^b^	16.9 ± 0.8 ^c^	5.4 ± 0.2 ^a^	15.9 ± 0.6 ^b^	6.6 ± 0.5 ^a^	15.2 ± 0.5 ^b^	25.1 ± 0.3 ^c^	13.6 ± 0.0 ^a^
16	(all-*E*)-β-cryptoxanthin	43.1 ± 1.1 ^b^	418.9 ± 1.5 ^c^	42.5 ± 2.8 ^a^	441.5 ± 1.0 ^b^	40.5 ± 1.4 ^a^	579.3 ± 2.7 ^d^	160.9 ± 2.2 ^c^	184.4 ± 0.4 ^a^
17	α-carotene-5,6-epoxide	2.6 ± 0.3 ^a^	n.d ^a^	5.5 ± 0.3 ^a^	n.d ^a^	8.5 ± 0.9 ^a^	n.d ^a^	31.9 ± 0.9 ^a^	n.d ^a^
18	(all-*E*)-luteoxanthin	n.d ^a^	n.d ^a^	n.d ^a^	n.d ^a^	n.d ^a^	n.d ^a^	64.7 ± 1.2 ^b^	n.d ^a^
19	(13*Z*)-α-carotene	14.0 ± 0.1 ^b^	12.8 ± 0.0 ^b^	13.5 ± 0.3 ^a^	12.7 ± 0.0 ^a^	14.3 ± 1.1 ^ab^	12.5 ± 0.2 ^a^	33.3 ± 0.6 ^c^	31.6 ± 0.8 ^c^
20	(13*Z*)-β-carotene	3.9 ± 0.1 ^a^	3.1 ± 0.1 ^a^	5.1 ± 0.2 ^a^	4.4 ± 0.2 ^a^	4.9 ± 0.1 ^a^	4.2 ± 0.1 ^a^	17.0 ± 0.2 ^b^	15.6 ± 0.8 ^b^
21	(all-*E*)-violaxanthin laurate	11.3 ± 1.2 ^ab^	n.d ^a^	8.1 ± 2.3 ^a^	n.d ^a^	20.2 ± 1.9 ^b^	n.d ^a^	89.5 ± 1.1 ^c^	n.d ^a^
22	β-cryptoxanthin-5,8-epoxide	7.7 ± 0.7 ^b^	n.d ^a^	3.2 ± 0.1 ^a^	n.d ^a^	14.1 ± 1.0 ^c^	n.d ^a^	57.8 ± 0.4 ^d^	46.8 ± 0.2 ^b^
23	(all-*E*)-ζ-carotene	4.2 ± 0.3 ^b^	1.6 ± 0.5 ^b^	n.d ^a^	n.d ^a^	55.7 ± 0.7 ^c^	32.9 ± 0.3 ^c^	77.5 ± 0.6 ^d^	75.1 ± 0.6 ^d^
24	β-cryptoxanthin-5,8’-epoxide	28.6 ± 0.3 ^b^	13.4 ± 0.2 ^a^	13.1 ± 1.8 ^a^	11.3 ± 0.2 ^a^	23.3 ± 1.2 ^a^	13.4 ± 1.9 ^a^	48.0 ± 3.5 ^b^	38.5 ± 2.8 ^b^
25	(all-*E*)-α-carotene	75.0 ± 0.0 ^c^	62.1 ± 0.1 ^c^	74.7 ± 0.5 ^a^	73.4 ± 0.1 ^a^	79.2 ± 0.5 ^ab^	72.5 ± 0.7 ^a^	95.9 ± 5.2 ^b^	93.2 ± 2.3 ^b^
26	(9*Z*)-α-carotene	3.2 ± 0.2 ^b^	2.4 ± 0.1 ^b^	n.d ^a^	n.d ^a^	4.1 ± 0.2 ^b^	3.3 ± 0.3 ^b^	10.3 ± 0.5 ^c^	8.7 ± 0.6 ^c^
27	(9*Z*)-violaxanthin laurate	50.9 ± 2.5 ^b^	n.d ^a^	n.d ^a^	n.d ^a^	59.0 ± 1.3 ^b^	n.d ^a^	107.5 ± 4.2 ^c^	n.d ^a^
28	(all-*E*)-lutein-3-*O*-myristate	168.7 ± 0.1 ^a^	n.d ^a^	210.7 ± 0.6 ^a^	57.4 ± 3.1 ^c^	261.6 ± 4.8 ^c^	35.1 ± 1.6 ^b^	278.0 ± 2.9 ^b^	n.d ^a^
29	(all-*E*)-β-carotene	164.5 ± 6.6 ^c^	159.2 ± 0.7 ^c^	120.3 ± 2.3 ^a^	132.9 ± 3.8 ^a^	170.3 ± 8.6 ^b^	166.5 ± 1.4 ^b^	405.2 ± 10.7 ^d^	337.5 ± 14.3 ^d^
30	(9Z)-β-carotene	5.9 ± 0.1 ^b^	5.1 ± 0.1 ^b^	5.2 ± 0.1 ^b^	6.4 ± 0.1 ^b^	n.d ^a^	n.d ^a^	25.8 ± 0.7 ^c^	21.3 ± 1.4 ^c^
31	(all-*E*)-violaxanthin dimyristate	36.0 ± 2.6 ^a^	n.d ^a^	41.3 ± 2.0 ^a^	n.d ^a^	48.1 ± 2.6 ^a^	n.d ^a^	97.5 ± 1.6 ^b^	n.d ^a^
32	(all-*E*)-antheraxanthin myristate palmitate	43.2 ± 1.6 ^b^	n.d ^a^	43.4 ± 0.4 ^a^	n.d ^a^	52.6 ± 2.4 ^b^	n.d ^a^	70.4 ± 3.3 ^c^	n.d ^a^
33	(all-*E*)-violaxanthin palmitate	6.8 ± 0.7 ^a^	n.d ^a^	10.7 ± 1.0 ^a^	n.d ^a^	20.2 ± 0.6 ^b^	n.d ^a^	46.0 ± 1.0 ^c^	n.d ^a^
34	(9*Z*)-neoxanthin dibutyrate	7.5 ± 0.3 ^a^	n.d ^a^	15.6 ± 0.6 ^b^	n.d ^a^	27.6 ± 2.3 ^c^	n.d ^a^	52.8 ± 1.8 ^d^	n.d ^a^
35	(all-*E*)-β-cryptoxanthin caprate	81.8 ± 1.5 ^d^	n.d ^a^	69.7 ± 2.1 ^a^	n.d ^a^	90.4 ± 2.2 ^c^	n.d ^a^	91.6 ± 1.2 ^b^	14.0 ± 0.3 ^b^
36	(9*Z*)-violaxanthin myristate palmitate	n.d ^a^	n.d ^a^	9.9 ± 0.2 ^b^	n.d ^a^	12.0 ± 0.4 ^c^	n.d ^a^	22.7 ± 0.5 ^d^	n.d ^a^
37	(all-*E*)-lutein dimyristate	60.3 ± 4.0 ^b^	n.d ^a^	61.6 ± 4.0 ^a^	n.d ^a^	79.7 ± 1.7 ^a^	n.d ^a^	92.1 ± 5.39 ^a^	n.d ^a^
38	(all-*E*)-β-cryptoxanthin laurate	174.9 ± 10.4 ^b^	n.d ^a^	167.8 ± 3.5 ^a^	n.d ^a^	223.2 ± 8.2 ^b^	n.d ^a^	281.7 ± 1.8 ^b^	n.d ^a^
39	(all-*E*)-antheraxanthin-3-*O* palmitate	n.d ^a^	n.d ^a^	n.d ^a^	n.d ^a^	n.d ^a^	n.d ^a^	56.6 ± 1.0 ^b^	n.d ^a^
40	(all-*E*)-antheraxanthin laurate myristate	21.9 ± 2.2 ^b^	8.2 ± 0.6 ^b^	17.2 ± 1.8 ^a^	n.d ^a^	16.7 ± 0.9 ^a^	n.d ^a^	36.5 ± 1.9 ^b^	n.d ^a^
41	(all-*E*)-β-cryptoxanthin myristate	18.6 ± 1.6 ^b^	4.6 ± 0.3 ^b^	12.8 ± 0.2 ^a^	n.d ^a^	21.3 ± 1.1 ^b^	22.6 ± 0.4 ^c^	28.3 ± 0.6 ^b^	n.d ^a^
42	(*Z*)-lycopene isomer 1	13.8 ± 0.6 ^b^	12.0 ± 0.5 ^b^	11.6 ± 0.5 ^a^	10.8 ± 0.4 ^a^	23.2 ± 1.8 ^c^	19.1 ± 1.3 ^c^	28.0 ± 0.6 ^c^	29.1 ± 1.0 ^d^
43	(all-*E*)-β-cryptoxanthin palmitate	13.9 ± 0.4 ^c^	5.5 ± 0.0 ^c^	5.2 ± 0.2 ^a^	n.d ^a^	10.0 ± 0.2 ^b^	10.7 ± 1.2 ^d^	20.6 ± 0.1 ^c^	4.2 ± 0.2 ^b^
44	(13*Z*)-lycopene isomer 2	112.6 ± 0.1 ^a^	98.3 ± 2.7 ^a^	194.3 ± 3.4 ^b^	189.0 ± 4.4 ^b^	198.1 ± 6.0 ^b^	203.7 ± 8.8 ^b^	256.9 ± 8.3 ^c^	141.9 ± 2.5 ^a^
45	(13’*Z*)-lycopene isomer 3	22.4 ± 0.2 ^a^	16.3 ± 0.3 ^b^	24.7 ± 0.5 ^a^	21.5 ± 0.6 ^b^	28.5 ± 1.4 ^a^	30.2 ± 1.9 ^c^	43.9 ± 1.7 ^b^	14.3 ± 0.4 ^a^
46	(9*Z*)-lycopene isomer 4	26.3 ± 0.5 ^a^	22.1 ± 0.7 ^b^	21.4 ± 2.0 ^a^	20.7 ± 0.4 ^a^	30.4 ± 2.3 ^a^	33.1 ± 1.2 ^c^	22.0 ± 0.9 ^a^	55.8 ± 0.6 ^d^
47	(9’*Z*)-lycopene isomer 5	12.3 ± 0.3 ^a^	9.7 ± 0.7 ^a^	21.0 ± 1.1 ^b^	21.3 ± 0.3 ^c^	18.2 ± 0.5 ^a^	16.6 ± 0.1 ^b^	27.8 ± 0.9 ^c^	14.3 ± 0.5 ^a^
48	(all-*E*)-lycopene	378.2 ± 5.4 ^c^	342.9 ± 4.3 ^c^	328.4 ± 2.0 ^a^	320.3 ± 2.9 ^a^	421.2 ± 1.2 ^b^	401.9 ± 7.6 ^b^	496.7 ± 1.8 ^b^	459.5 ± 3.2 ^b^
49	(*Z*)-lycopene isomer 6	22.6 ± 0.6 ^b^	20.4 ± 0.6 ^b^	16.1 ± 0.3 ^a^	18.7 ± 0.4 ^a^	36.5 ± 1.5 ^c^	33.3 ± 1.3 ^c^	85.7 ± 2.1 ^d^	46.9 ± 1.1 ^d^
	total free xanthophylls	109.0. ± 4.0 ^a^	1204.5 ± 33.7 ^a^	84.6 ± 6.1 ^a^	1257.0 ± 17.5 ^a^	120.5 ± 6.4 ^a^	1417.6 ± 37.8 ^b^	879.0 ± 25.7 ^b^	1566.5 ± 18.0 ^c^
	total xanthophyll esters	695.8 ± 29.5 ^a^	18.2 ± 1.0 ^a^	673.9 ± 19.8 ^a^	57.4 ± 3.1 ^b^	942.5 ± 30.7 ^b^	68.5 ± 7.2 ^b^	1371.7 ± 24.6 ^c^	84.0 ± 2.6 ^c^
	total hydrocarbon carotenoids	859.0 ± 15.4 ^a^	767.9 ± 11.3 ^a^	836.2 ± 14.0 ^a^	832.2 ± 13.6 ^b^	1084.6 ± 26.6 ^b^	1029.9 ± 25.2 ^c^	1657.7 ± 33.7 ^c^	1344.8 ± 30.0 ^d^
	total carotenoids	1663.7 ± 48.9 ^a^	1990.7 ± 46.1 ^a^	1594.7 ± 39.7 ^a^	2146.6 ± 34.2 ^b^	2147.5 ± 63.7 ^b^	2515.9 ± 70.2 ^c^	3908.4 ± 84.0 ^c^	2995.3 ± 50.6 ^d^
	RAE ^4^	22.7 ± 0.7 ^b^	25.7 ± 0.1 ^a^	18.0 ± 0.5 ^a^	22.6 ± 0.1 ^a^	23.4 ± 0.9 ^b^	25.5 ± 0.2 ^a^	41.1 ± 0.9 ^c^	43.3 ± 1.7 ^a^

tr., traces; n.d., not detected (detection limit: 0.08 µg/g). ^1^ Results are expressed as the mean ± standard deviation of duplicate analysis (*n* = 2) of samples from freeze-dried papaya pulp. Different superscript letters indicate statistically significant differences (*p* ≤ 0.05). ^2^ Numbers correspond with the HPLC-DAD chromatogram peaks (Table 2). ^3^ Internal standard. ^4^ Retinol activity equivalent calculated according to guidelines of the United States (US) Institute of Medicine [27].

**Table 4:** Carotenoid content ^1^ (μg/100 g fresh weight ± standard deviation) and retinol activity equivalent (RAE) of direct and saponified extracts from papaya (*Carica papaya* L.) peels of Sweet Mary, Alicia, Eksotika, and Maradol varieties.

No.^2^	Carotenoid Compound	cv. Sweet Mary		cv. Alicia		cv. Eksotika		cv. Maradol	
		Direct Extract (C)	Saponified Extract (SAP)	Direct Extract (C)	Saponified Extract (SAP)	Direct Extract (C)	Saponified Extract (SAP)	Direct Extract (C)	Saponified Extract (SAP)
1	(13*Z*)-violaxanthin	36.7 ± 2.4 ^b^	152.5 ± 9.4 ^a^	24.0 ± 1.3 ^a^	275.2 ± 12.6 ^c^	76.8 ± 9.8 ^d^	165.9 ± 8.1 ^a^	49.9 ± 0.3 ^c^	160.2 ± 4.2 ^b^
2	(all-*E*)-violaxanthin	186.5 ± 0.3 ^a^	441.4 ± 15.9 ^b^	116.5 ± 1.4 ^a^	411.6 ± 8.2 ^a^	284.0 ± 2.3 ^b^	349.0 ± 22.5 ^a^	142.9 ± 0.3 ^a^	421 ± 12.4 ^b^
3	not identified 1	tr	tr.	tr.	tr.	tr.	tr.	tr.	tr.
4	(9*Z*)-neoxanthin	7.8 ± 0.7 ^b^	472.0 ± 4.3 ^d^	n.d ^a^	62.4 ± 4.8 ^b^	62.4 ± 4.8 ^c^	124.5 ± 1.7 ^a^	4.8 ± 0.6 ^ab^	162.5 ± 5.6 ^c^
5	(all-*E*)-neoxanthin	32.4 ± 0.9 ^a^	152.4 ± 4.1 ^c^	36.0 ± 2.2 ^b^	123.3 ± 0.9 ^a^	38.7 ± 2.6 ^c^	148.3 ± 9.9 ^b^	46.0 ± 0.4 ^bc^	151.2 ± 2.7 ^c^
6	(all-*E*)-lutein	922.5 ± 9.1 ^b^	782.9 ± 6.2 ^a^	1381.1 ± 3.9 ^c^	1238.4 ± 10.1 ^a^	1094.1 ± 5.2 ^c^	1499.9 ± 34.9 ^a^	492.4 ± 1.2 ^a^	852.6 ± 6.1 ^a^
7	(all-*E*)-zeaxanthin	44.6 ± 3.2 ^a^	120.0 ± 8.4 ^a^	127.1 ± 10.1 ^c^	195.3 ± 0.8 ^b^	81.3 ± 2.2 ^b^	133.9 ± 2.3 ^a^	59.0 ± 1.9 ^a^	129.4 ± 2.3 ^a^
8	lutein-5,6-epoxide	n.d ^a^	273.5 ± 7.3 ^b^	n.d ^a^	n.d ^a^	n.d ^a^	n.d ^a^	38.8 ± 1.0 ^b^	160.0 ± 6.0 ^b^
9	(all-*E*)-antheraxanthin	25.2 ± 1.1 ^a^	116.9 ± 9.6 ^a^	20.9 ± 1.7 ^a^	139.7 ± 5.1 ^b^	40.0 ± 3.1 ^b^	102.6 ± 5.8 ^b^	64.7 ± 5.5 ^c^	140.9 ± 7.6 ^b^
10	(9*Z*)-violaxanthin	20.3 ± 0.1 ^c^	n.d ^a^	7.8 ± 0.2 ^b^	n.d ^a^	n.d ^a^	n.d ^a^	n.d ^a^	n.d ^a^
11	(all-*E*)-β-apo-caroten-8 al (IS^3^)	139.7 ± 0.9 ^b^	138.7 ± 0.4 ^b^	130.4 ± 0.9 ^b^	133.3 ± 0.6 ^a^	119.9 ± 13.4 ^b^	110.0 ± 4.4 ^a^	147.0 ± 0.6 ^a^	141.2 ± 2.3 ^b^
12	β-cryptoxanthin-5,6-epoxide	5.1 ± 0.5 ^b^	71.3 ± 3.0 ^b^	n.d ^a^	89.9 ± 0.2 ^b^	64.5 ± 4.8 ^d^	32.8 ± 1.0 ^a^	12.1 ± 0.2 ^c^	36.2 ± 0.9 ^a^
13	(9*Z*)-α-cryptoxanthin	10.9 ± 0.6 ^b^	7.2 ± 0.1 ^b^	n.d ^a^	21.5 ± 0.2 ^c^	n.d ^a^	n.d ^a^	18.3 ± 0.0 ^c^	27.9 ± 0.3 ^c^
14	not identified 2	tr.	n.d	n.d	tr.	n.d	tr.	n.d	tr.
15	(all-*E*)-α-cryptoxanthin	3.5 ± 0.5 ^b^	29.8 ± 3.2 ^b^	n.d ^a^	30.7 ± 0.3 ^b^	n.d ^a^	8.3 ± 0.7 ^a^	7.7 ± 0.0 ^c^	12 ± 0.2 ^a^
16	(all-*E*)-β-cryptoxanthin	65.6 ± 1.4 ^a^	188.9 ± 9.0 ^b^	68.5 ± 3.9 ^a^	170.7 ± 1.4 ^a^	70.7 ± 2.1 ^a^	183.8 ± 14.1 ^c^	88.2 ± 0.7 ^a^	197.8 ± 8.2 ^d^
17	α-carotene-5,6-epoxide	32.7 ± 0.1 ^b^	23.8 ± 0.2 ^a^	8.2 ± 1.2 ^a^	42.8 ± 3.1 ^b^	7.2 ± 1.4 ^a^	22.5 ± 1.4 ^a^	34.9 ± 0.4 ^b^	32.1 ± 1.0 ^b^
18	(all-*E*)-luteoxanthin	103.2 ± 5.1 ^c^	195.3 ± 5.2 ^c^	n.d ^a^	n.d ^a^	n.d ^a^	16.2 ± 0.0 ^b^	36.3 ± 1.2 ^b^	34.7 ± 0.7 ^b^
19	(13*Z*)-α-carotene	89.6 ± 0.8 ^c^	64.8 ± 0.8 ^b^	22.7 ± 1.6 ^b^	n.d ^a^	13.6 ± 1.0 ^a^	n.d ^a^	18.2 ± 0.8 ^a^	17.8 ± 0.7 ^b^
20	(13*Z*)-β-carotene	9.8 ± 0.7 ^a^	6.6 ± 0.1 ^a^	47.5 ± 1.5 ^c^	46.2 ± 3.2 ^c^	20.4 ± 0.9 ^b^	15.1 ± 0.4 ^b^	29.5 ± 0.2 ^b^	20.4 ± 0.2 ^b^
21	(all-*E*)-violaxanthin laurate	141.3 ± 0.1 ^c^	n.d ^a^	4.7 ± 0.1 ^a^	n.d ^a^	31.9 ± 1.3 ^b^	n.d ^a^	39.8 ± 1.9 ^b^	n.d ^a^
22	β-cryptoxanthin-5,8-epoxide	25.7 ± 0.9 ^c^	n.d ^a^	16.6 ± 0.2 ^b^	n.d ^a^	43.9 ± 2.2 ^d^	70.7 ± 6.4 ^b^	n.d ^a^	n.d ^a^
23	(all-*E*)-ζ-carotene	7.7 ± 2.0 ^a^	5.7 ± 0.7 ^b^	90.2 ± 3.9 ^b^	58.4 ± 1.9 ^d^	109.7 ± 11.4 ^c^	33.9 ± 0.1 ^c^	n.d ^a^	n.d ^a^
24	β-cryptoxanthin-5,8’-epoxide	35.6 ± 1.0 ^c^	n.d ^a^	n.d ^a^	n.d ^a^	35.5 ± 3.2 ^d^	32.4 ± 1.3 ^c^	11.9 ± 0.1 ^b^	20.8 ± 0.3 ^b^
25	(all-*E*)-α-carotene	22.5 ± 1.4 ^b^	14.8 ± 0.2 ^b^	n.d ^a^	n.d ^a^	40.7 ± 1.3 ^c^	n.d ^a^	105.1 ± 0.4 ^d^	95.6 ± 2.1 ^c^
26	(9*Z*)-α-carotene	9.8 ± 1.0 ^b^	6.5 ± 0.1 ^b^	n.d ^a^	n.d ^a^	6.1 ± 0.4 ^b^	n.d ^a^	14.7 ± 0.3 ^c^	12.5 ± 0.5 ^c^
27	(9*Z*)-violaxanthin laurate	19.9 ± 1.1 ^b^	n.d ^a^	n.d ^a^	n.d ^a^	69.4 ± 4.2 ^c^	n.d ^a^	141.7 ± 0.7 ^d^	n.d ^a^
28	(all-*E*)-lutein-3-*O*-myristate	117.5 ± 2.4 ^c^	n.d ^a^	70.9 ± 3.3 ^b^	n.d ^a^	n.d ^a^	n.d ^a^	229.0 ± 0.4 ^d^	n.d ^a^
29	(all-*E*)-β-carotene	233.2 ± 5.4 ^b^	200.1 ± 2.6 ^b^	157.1 ± 5.9 ^a^	144.0 ± 4.1 ^a^	223.6 ± 9.5 ^c^	207.8 ± 13.8 ^c^	251.4 ± 0.8 ^b^	239.8 ± 1.3 ^d^
30	(9*Z*)-β-carotene	n.d ^a^	n.d ^a^	n.d ^a^	n.d ^a^	n.d ^a^	n.d ^a^	11.1 ± 1.0 ^c^	8.1 ± 0.1 ^b^
31	(all-*E*)-violaxanthin dimyristate	28.1 ± 0.8 ^c^	n.d ^a^	11.8 ± 1.4 ^b^	n.d ^a^	n.d ^a^	n.d ^a^	240.2 ± 8.0 ^d^	n.d ^a^
32	(all-*E*)-antheraxanthin myristate palmitate	146.3 ± 6.2 ^c^	n.d ^a^	95.4 ± 2.7 ^b^	52.1 ± 2.8 ^b^	125.6 ± 1.7 ^c^	n.d ^a^	70.2 ± 1.0 ^a^	50.4 ± 4.6 ^b^
33	(all-*E*)-violaxanthin palmitate	53.7 ± 1.5 ^d^	n.d ^a^	29.6 ± 3.2 ^c^	n.d ^a^	8.9 ± 0.4 ^b^	n.d ^a^	n.d ^a^	n.d ^a^
34	(9*Z*)-neoxanthin dibutyrate	44.3 ± 2.2 ^c^	n.d ^a^	16.5 ± 2.8 ^b^	n.d ^a^	6.7 ± 0.2 ^a^	n.d ^a^	38.7 ± 2.5 ^c^	n.d ^a^
35	(all-*E*)-β-cryptoxanthin caprate	54.1 ± 0.1 ^b^	n.d ^a^	28.9 ± 2.7 ^a^	n.d ^a^	45.2 ± 3.1 ^b^	n.d ^a^	69.0 ± 0.4 ^c^	n.d ^a^
36	(9*Z*)-violaxanthin myristate palmitate	28.1 ± 0.1 ^b^	n.d ^a^	12.4 ± 1.7 ^a^	n.d ^a^	11.1 ± 1.0 ^a^	n.d ^a^	47.0 ± 4.0 ^c^	n.d ^a^
37	(all-*E*)-lutein dimyristate	49.0 ± 2.3 ^a^	n.d ^a^	39.9 ± 4.5 ^a^	n.d ^a^	39.1 ± 3.1 ^a^	n.d ^a^	91.8 ± 0.2 ^b^	n.d ^a^
38	(all-*E*)-β-cryptoxanthin laurate	74.4 ± 5.7 ^b^	n.d ^a^	66.8 ± 0.0 ^a^	n.d ^a^	64.0 ± 0.4 ^a^	n.d ^a^	194.9 ± 8.0 ^c^	n.d ^a^
39	(all-*E*)-antheraxanthin-3-*O*-palmitate	74.7 ± 3.2 ^c^	n.d ^a^	23.3 ± 0.4 ^a^	n.d ^a^	120.2 ± 7.1 ^c^	n.d ^a^	54.8 ± 0.2 ^b^	n.d ^a^
40	(all-*E*)-antheraxanthin laurate myristate	36.9 ± 2.0 ^b^	n.d ^a^	24.0 ± 0.2 ^a^	n.d ^a^	26.3 ± 1.0 ^a^	n.d ^a^	61.5 ± 0.7 ^c^	42.6 ± 5.4 ^a^
41	(all-*E*)-β-cryptoxanthin myristate	35.6 ± 1.7 ^b^	n.d ^a^	24.4 ± 2.0 ^a^	n.d ^a^	22.7 ± 1.8 ^a^	4.4 ± 0.2 ^b^	57.4 ± 0.3 ^c^	n.d ^a^
42	(*Z*)-lycopene isomer 1	17.6 ± 0.2 ^b^	12.8 ± 0.3 ^b^	n.d ^a^	n.d ^a^	21.4 ± 1.3 ^b^	15.6 ± 1.1 ^c^	25.3 ± 1.0 ^b^	22.5 ± 0.3 ^d^
43	(all-*E*)-β-cryptoxanthin palmitate	n.d ^a^	n.d ^a^	n.d ^a^	n.d ^a^	n.d ^a^	n.d ^a^	11.2 ± 0.3 ^b^	n.d ^a^
44	(13*Z*)-lycopene isomer 2	17.9 ± 1.0 ^a^	15.9 ± 0.4 ^a^	41.5 ± 0.4 ^b^	41.1 ± 1.3 ^b^	57.7 ± 2.0 ^c^	41.8 ± 1.2 ^c^	258.2 ± 1.1 ^d^	254.3 ± 1.2 ^d^
45	(13’*Z*)-lycopene isomer 3	n.d ^a^	n.d ^a^	n.d ^a^	n.d ^a^	n.d ^a^	n.d ^a^	20.4 ± 1.2 ^b^	18.9 ± 1.1 ^b^
46	(9*Z*)-lycopene isomer 4	22.0 ± 0.2 ^a^	15.2 ± 0.2 ^a^	37.0 ± 0.8 ^b^	35.6 ± 0.5 ^b^	50.8 ± 2.1 ^c^	35.9 ± 1.2 ^c^	67.7 ± 1.5 ^c^	67.1 ± 0.8 ^d^
47	(9’*Z*)-lycopene isomer 5	n.d ^a^	n.d ^a^	n.d ^a^	n.d ^a^	11.5 ± 0.9 ^b^	8.0 ± 0.5 ^b^	20.7 ± 2.1 ^c^	15.2 ± 0.4 ^c^
48	(all-*E*)-lycopene	306.6 ± 3.9 ^b^	251.3 ± 0.2 ^b^	230.0 ± 2.2 ^a^	231.3 ± 1.1 ^a^	318.8 ± 26.8 ^c^	238.4 ± 20.4 ^c^	441.0 ± 2.0 ^d^	420.5 ± 12.4 ^d^
49	(*Z*)-lycopene isomer 6	41.8 ± 1.5 ^a^	32.5 ± 0.2 ^a^	48.9 ± 1.4 ^b^	45.9 ± 0.6 ^a^	58.2 ± 2.9 ^c^	44.2 ± 3.7 ^b^	50.0 ± 1.1 ^a^	42.6 ± 3.8 ^b^
	total free xanthophylls	1525.6 ± 28.0 ^b^	3010.9 ± 95.2 ^b^	1806.7 ± 29.3 ^c^	2910.2 ± 51.3 ^b^	1899.1 ± 43.7 ^c^	2868.1 ± 224.6 ^b^	1073.1 ± 13.4 ^a^	2507.2 ± 83.2 ^a^
	total xanthophyll esters	903.9 ± 29.6 ^b^	0.0 ± 0.0 ^a^	448.7 ± 25.0 ^a^	52.1 ± 2.8 ^b^	571.1 ± 29.6 ^a^	4.4 ± 0.3 ^a^	1347.2 ± 20.8 ^c^	93.0 ± 10.0 ^b^
	total hydrocarbon carotenoids	811.2 ± 16.6 ^b^	649.9 ± 5.9 ^a^	675.0 ± 17.6 ^a^	645.2 ± 15.8 ^a^	964.5 ± 75.4 ^a^	663.3 ± 43.8 ^a^	1348.3 ± 14.1 ^c^	1222.4 ± 29.7 ^b^
	total carotenoids	3240.8 ± 74.3 ^b^	3660.8 ± 101.1 ^a^	2930.4 ± 71.8 ^a^	3607.5 ± 69.9 ^a^	3434.7 ± 148.7 ^a^	3535.8 ± 268.7 ^a^	3768.6 ± 48.3 ^c^	3822.6 ± 122.9 ^a^
	RAE^4^	36.7 ± 3.2 ^b^	28.2 ± 1.5 ^ab^	28.7 ± 0.6 ^a^	24.7 ± 0.5 ^a^	36.7 ± 1.6 ^a^	31.8 ± 3.3 ^ab^	34.0 ± 0.4 ^ab^	35.7 ± 0.8 ^b^

tr., traces; n.d., not detected (detection limit: 0.08 µg/g). ^1^ Results are expressed as the mean ± standard deviation of duplicate analysis (*n* = 2) of samples from freeze-dried papaya pulp. Different superscript letters indicate statistically significant differences (*p* ≤ 0.05). ^2^ Numbers correspond with the HPLC-DAD chromatogram peaks (Table 2). ^3^ Internal standard. ^4^ Retinol activity equivalent calculated according to guidelines of the US Institute of Medicine [27].

## Supplementary Materials

The following are available online at https://www.mdpi.com/2304-8158/10/2/434/s1: Figure S1. C_30_ reversed-phase chromatogram of carotenoids of direct (A) or saponified extracts (B) obtained from pulp (PU) and peel (PE) of papaya fruits (*Carica papaya*, L.) cv. Alicia, detected at 450 nm. Peak identities are shown in Table 2; Figure S2. C_30_ reversed-phase chromatogram of carotenoids of direct (A) or saponified extracts (B) obtained from pulp (PU) and peel (PE) of papaya fruits (*Carica papaya*, L.) cv. Eksotika, detected at 450 nm. Peak identities are shown in Table 2; Figure S3. C_30_ reversed-phase chromatogram of carotenoids of direct (A) or saponified extracts (B) obtained from pulp (PU) and peel (PE) of papaya fruits (*Carica papaya*, L.) cv. Maradol, detected at 450 nm. Peak identities are shown in Table 2; Figure S4. Carotenoid proportion (%) of free xanthophylls, hydrocarbon carotenoids, and xanthophyll esters (A) in direct extracts (crude) and (B) in saponified extracts of papaya pulps of cv. Sweet Mary, cv. Alicia, cv. Eksotika, and cv. Maradol; Figure S5. Carotenoid proportion (%) of free xanthophylls, hydrocarbon carotenoids, and xanthophyll esters (A) in direct extracts (crude) and (B) in saponified extracts of papaya peels of cv. Sweet Mary, cv. Alicia, cv. Eksotika, and cv. Maradol; Figure S6. Carotenoid proportion (%) of carotenoid species in direct extracts (crude) and in saponified extracts of papaya (*Carica papaya* L.) (A) pulp and (B) peels of cv. Sweet Mary, cv. Alicia, cv. Eksotika, and cv. Maradol.

## References

[1] Sandoval K.V., Ávila D.D., Gracia T.J.H. (2017). Estudio del mercado de papaya mexicana: Un análisis de su competitividad (2001–2015). SUMNEG.

[2] Cabrera J.A., Raya V., Lobo M.G., Ritter A. Effect of climate conditions on growth and production of hydroponic papaya crops in the Canary Islands. Proceedings of the XI International Symposium on Protected Cultivation in Mild Winter Climates and I International Symposium on Nettings and Screens in Horticulture.

[3] Hueso J.J., Salinas I., Pinillos V., Cuevas J. Papaya greenhouse cultivation in south-east Spain. Proceedings of the V International Symposium on Papaya.

[4] Ramona C., Ana B., Mihai C., Stãnicã F. (2017). *Carica papaya* L. cultivated in greenhouse conditions. J. Hortic. Sci. Biotechnol..

[5] Ramos-Parra P.A., García-Salinas C., Rodríguez-López C.E., García N., García-Rivas G., Hernández-Brenes C., de la Garza R.I.D. (2019). High hydrostatic pressure treatments trigger the novo carotenoid biosynthesis in papaya fruit (*Carica papaya* cv. Maradol). Food Chem..

[6] Addai Z.R., Abdullah A., Mutalib S.A., Musa K.H., Douqan E.M. (2013). Antioxidant activity and physicochemical properties of mature papaya fruit (*Carica papaya* L. cv. Eksotika). Adv. J. Food Sci. Technol..

[7] Sancho L.E.G.G., Yahia E.M., González-Aguilar G.A. (2011). Identification and quantification of phenols, carotenoids, and vitamin C from papaya (*Carica papaya* L., cv. Maradol) fruit determined by HPLC-DAD-MS/MS-ESI. Food Res. Int..

[8] Cano M.P., de Ancos B., Lobo G., Monreal M. (1996). Carotenoid pigments and colour of hermaphrodite and female papaya fruits (*Carica papaya* L.) cv. Sunrise during post-harvest ripening. J. Agric. Food Chem..

[9] Britton G. (1995). Structure and properties of carotenoids in relation to function. FASEB J..

[10] Bunea A., Socaciu C., Pintea A. (2014). Xanthophyll esters in fruits and vegetables. Not. Bot. Hort. Agrob..

[11] Ampomah-Dwamena C., Driedonks N., Lewis D., Shumskaya M., Chen X., Wurtzel E.T., Allan A.C. (2015). The Phytoene synthase gene family of apple (*Malus x domestica*) and its role in controlling fruit carotenoid content. BMC Plant. Biol..

[12] Lux P.E., Carle R., Zacarias L., Rodrigo M.J., Schweiggert R.M., Steingass C.B. (2019). Genuine carotenoid profiles in sweet orange [*Citrus sinensis* (L.) Osbeck cv. Navel] peel and pulp at different maturity stages. J. Agric. Food Chem..

[13] Petry F.C., Mercadante A.Z. (2016). Composition by LC-MS/MS of new carotenoid esters in mango and citrus. J. Agric. Food Chem..

[14] Petry F.C., Mercadante A.Z. (2017). Impact of *in vitro* digestion phases on the stability and bioaccessibility of carotenoids and their esters in mandarin pulps. Food Funct..

[15] Cano M.P., Gómez-Maqueo A., Fernández-López R., Welti-Chanes J., García-Cayuela T. (2019). Impact of high hydrostatic pressure and thermal treatment on the stability and bioaccessibility of carotenoid and carotenoid esters in astringent persimmon (*Diospyros kaki* Thunb, var. Rojo Brillante). Food Res. Int..

[16] Schweiggert R.M., Vargas E., Conrad J., Hempel J., Gras C.C., Ziegler J.U., Carle R. (2016). Carotenoids, carotenoid esters, and anthocyanins of yellow-, orange-, and red-peeled cashew apples (*Anacardium occidentale* L.). Food Chem..

[17] Gómez-Maqueo A., Bandino E., Hormaza J.I., Cano M.P. (2020). Characterization and the impact of *in vitro* simulated digestion on the stability and bioaccessibility of carotenoids and their esters in two Pouteria lucuma varieties. Food Chem..

[18] Schweiggert R.M., Carle R. (2017). Carotenoid deposition in plant and animal foods and its impact on bioavailability. Crit. Rev. Food Sci. Nutr..

[19] Ramos-Parra P.A., García-Salinas C., Díaz de la Garza R.I. (2013). Folate levels and polyglutamylation profiles of papaya (*Carica papaya* cv. Maradol) during fruit development and ripening. J. Agric. Food Chem..

[20] Plaza L., Colina C., de Ancos B., Sánchez-Moreno C., Cano M.P. (2012). Influence of ripening and astringency on carotenoid content of high-pressure treated persimmon fruit (*Diospyros kaki* L.). Food Chem..

[21] Breithaupt D.E., Wirt U., Bamedi A. (2002). Differentiation between lutein monoester regioisomers and detection of lutein diesters from marigold flowers (*Tagetes erecta* L.) and several fruits by liquid chromatography-mass spectrophotometry. J. Agric. Food Chem..

[22] De Faria A.F., De Rosso V.V., Mercadante A.Z. (2009). Carotenoid composition of jackfruit (*Artocarpus heterophyllus*), determined by HPLC-PDA MS/MS. Plant. Foods Hum. Nutr..

[23] De Rosso V.V., Mercadante A.Z. (2007). Identification and quantification of carotenoids, by HPLC-PDA-MS/MS, from Amazonian fruits. J. Agric. Food Chem..

[24] Mariutti L.R., Rodrigues E., Mercadante A.Z. (2013). Carotenoids from *Byrsonima crassifolia*: Identification, quantification and *in vitro* scavenging capacity against peroxyl radicals. J. Food Compos. Anal..

[25] Rodrigues D.B., Mercadante A.Z., Mariutti L.R.B. (2019). Marigold carotenoids: Much more than lutein esters. Food Res. Int..

[26] Van Breemen R.B., Dong L., Pajkovic N.D. (2012). Atmospheric pressure chemical ionization tandem mass spectrometry of carotenoids. Int. J. Mass Spectrom..

[27] Institute of Medicine (US) (2001). Panel on Micronutrients—Dietary Reference Intakes for Vitamin A, Vitamin K, Arsenic, Boron, Chromium, Copper, Iodine, Iron, Manganese, Molybdenum, Nickel, Silicon, Vanadium, and Zinc.

[28] Breithaupt D., Schwack W. (2000). Determination of free and bound carotenoids in paprika (*Capsicum annuum* L.) by LC/MS. Eur. Food Res. Technol..

[29] Molnár P., Martus Z., Szabolcs J., Körtvélyesi T. (1997). Kinetic studies on the thermal Z/E-isomerization of C40-carotenoids. J. Chem. Res. Synop..

[30] Melendez-Martinez A.J., Stinco C.M., Liu C., Wang X.D. (2013). A simple HPLC method for the comprehensive analysis of cis/trans (Z/E) geometrical isomers of carotenoids for nutritional studies. Food Chem..

[31] Cano M.P., Gómez-Maqueo A., Welti-Chanes J., García-Cayuela T. (2018). Characterization of carotenoid and carotenoid esters of astringent persimmon tissues (*Diospyros kaki* Thunb. var. Rojo brillante). Effects of thermal and high pressure non-thermal processing. Preprints.

[32] De Rosso V.V., Mercadante A.Z. (2005). Carotenoid composition of two Brazilian genotypes of acerola (*Malpighia punicifolia* L.) from two harvests. Food Res. Int..

[33] Chandrika U.G., Jansz E.R., Wickramasinghe S.N., Warnasuriya N.D. (2003). Carotenoids in yellow-and red-fleshed papaya (*Carica papaya* L). J. Sci. Food Agric..

[34] Schweiggert R.M., Steingass C.B., Esquivel P., Carle R. (2012). Chemical and morphological characterization of Costa Rican papaya (*Carica papaya* L.) hybrids and lines with particular focus on their genuine carotenoid profiles. J. Agric. Food Chem..

[35] Rivera-Pastrana D.M., Yahia E.M., González-Aguilar G.A. (2010). Phenolic and carotenoid profiles of papaya fruit (*Carica papaya* L.) and their contents under low temperature storage. J. Sci. Food Agric..

[36] Rodrigues D.B., Mariutti L.R.B., Mercadante A.Z. (2016). An *in vitro* digestion method adapted for carotenoids and carotenoid esters: Moving forward towards standardization. Food Funct..

[37] Britton G., Khachik F., Britton G., Pfander H., Liaaen-Jensen S. (2009). Carotenoids in food. Carotenoids.

[38] Becerra M.O., Contreras L.M., Lo M.H., Díaz J.M., Herrera G.C. (2020). Lutein as a functional food ingredient: Stability and bioavailability. J. Funct. Foods..

[39] Schweiggert R.M., Steingass C.B., Heller A., Esquivel P., Carle R. (2011). Characterization of chromoplasts and carotenoids of red-and yellow-fleshed papaya (*Carica papaya* L.). Planta.

[40] Shen Y.H., Yang F.Y., Lu B.G., Zhao W.W., Jiang T., Feng L., Ming R. (2019). Exploring the differential mechanisms of carotenoid biosynthesis in the yellow peel and red flesh of papaya. BMC Genom..

[41] Wall M.M. (2006). Ascorbic acid, vitamin A, and mineral composition of banana (*Musa* sp.) and papaya (*Carica papaya*) cultivars grown in Hawaii. J. Food Compos. Anal..

[42] Vasquez-Caicedo A.L., Heller A., Neidhart S., Carle R. (2006). Chromoplast morphology and β-carotene accumulation during postharvest ripening of mango Cv. ‘Tommy Atkins’. J. Agric. Food Chem..

[43] Cooperstone J.L., Ralston R.A., Riedl K.M., Haufe T.C., Schweiggert R.M., King S.A., Schwartz S.J. (2015). Enhanced bioavailability of lycopene when consumed as cis-isomers from tangerine compared to red tomato juice, a randomized, cross-over clinical trial. Mol. Nutr. Food Res..

[44] Schweiggert R.M., Kopec R.E., Villalobos-Gutierrez M.G., Högel J., Quesada S., Esquivel P., Carle R. (2014). Carotenoids are more bioavailable from papaya than from tomato and carrot in humans: A randomised cross-over study. Br. J. Nutr..

[45] Zaripheh S., Erdman J.W. (2002). Factors that influence the bioavailability of xanthopylls. J. Nutr..

[46] Amar I., Abraham A., Nissim G. (2013). Solubilization patterns of lutein and lutein esters in food grade nonionic microemulsions. J. Agric. Food Chem..

[47] Mariutti L.R., Mercadante A.Z. (2018). Carotenoid esters analysis and occurrence: What do we know so far?. Arch. Bichem. Biophys..

[48] Schweiggert R.M., Mezger D., Schimpf F., Steingass C.B., Carle R. (2012). Influence of chromoplast morphology on carotenoid bioaccessibility of carrot, mango, papaya, and tomato. Food Chem..

