# Supplementary material for: Carotenoid and Carotenoid Ester Profile and Their Deposition in Plastids in Fruits of New Papaya (Carica papaya L.) Varieties from the Canary Islands
Source: Foods. 2021 Feb 17;10(2):434. doi: 10.3390/foods10020434 (PMC7921962; doi:10.3390/foods10020434)
Supplement: Supplementary file 1 [file foods-10-00434-s001.pdf]

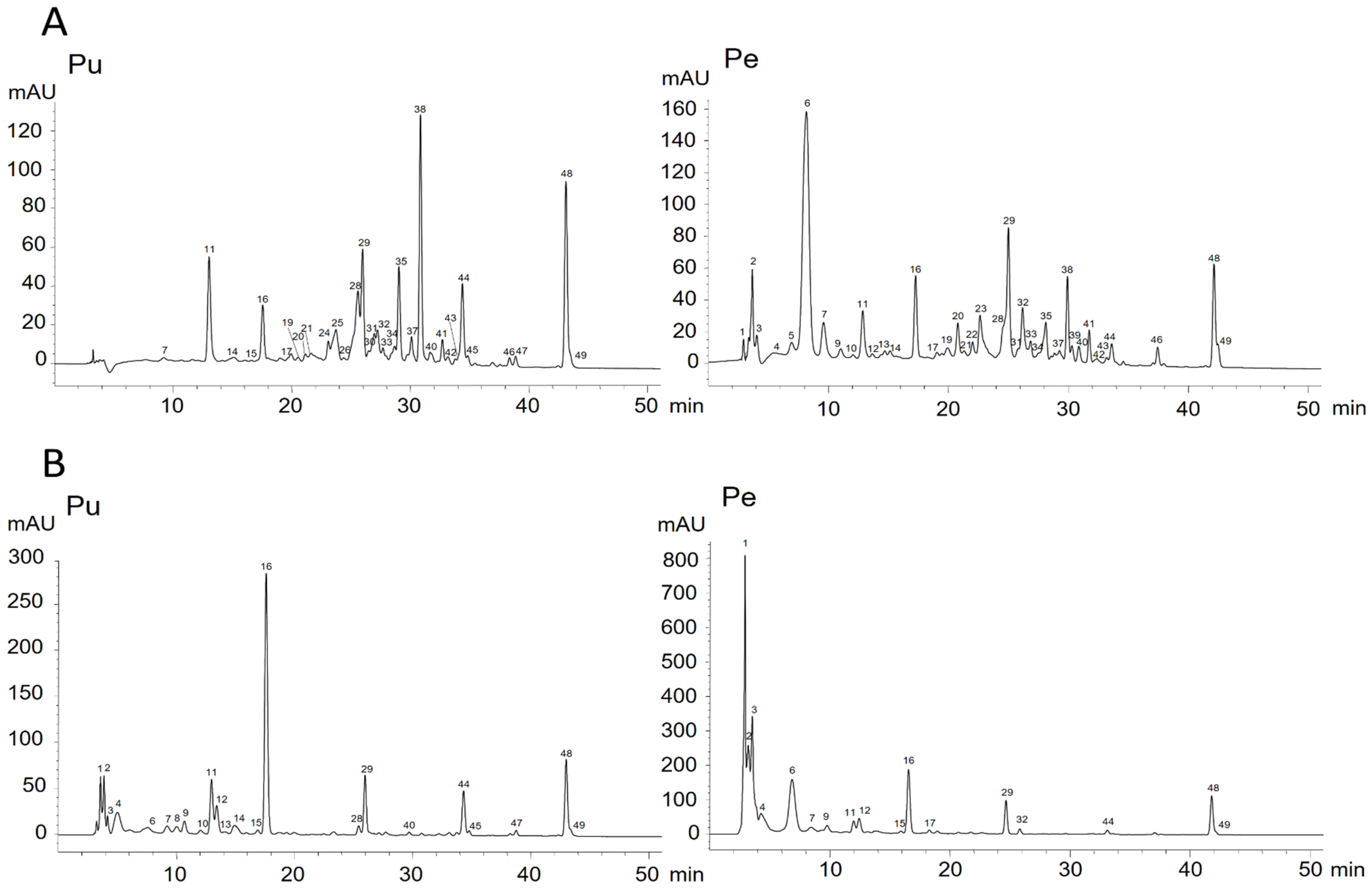

Figure S1. C_{30} reversed-phase chromatogram of carotenoids of direct (A) or saponified extracts (B) obtained from pulp (PU) and peel (PE) of papaya fruits (*Carica papaya*, L.) cv. Alicia, detected at 450 nm. Peak identities are showed in Table 2.

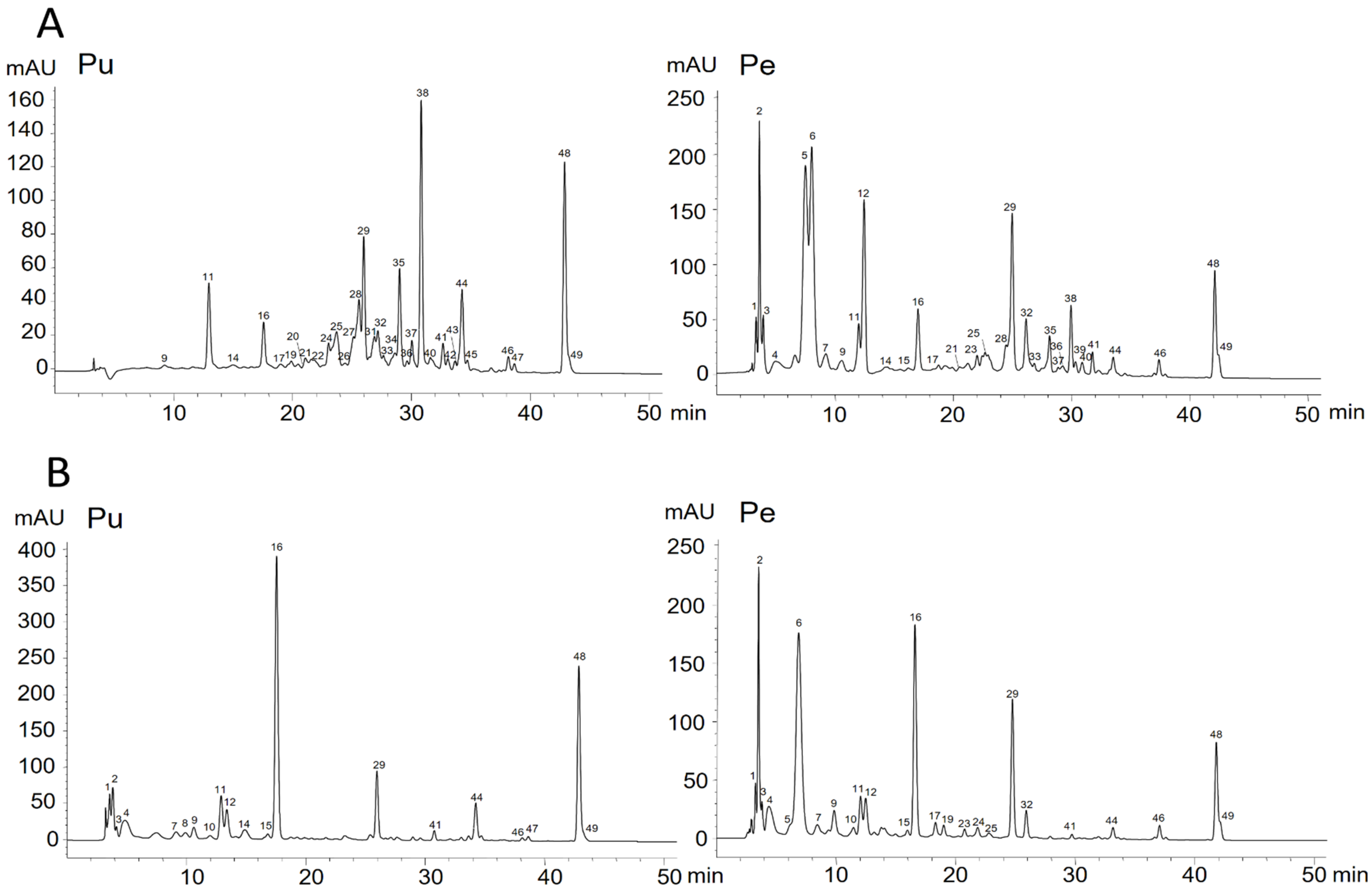

Figure S2. C_{30} reversed-phase chromatogram of carotenoids of direct (A) or saponified extracts (B) obtained from pulp (PU) and peel (PE) of papaya fruits (*Carica papaya*, L.) cv. Eksotika, detected at 450 nm. Peak identities are showed in Table 2.

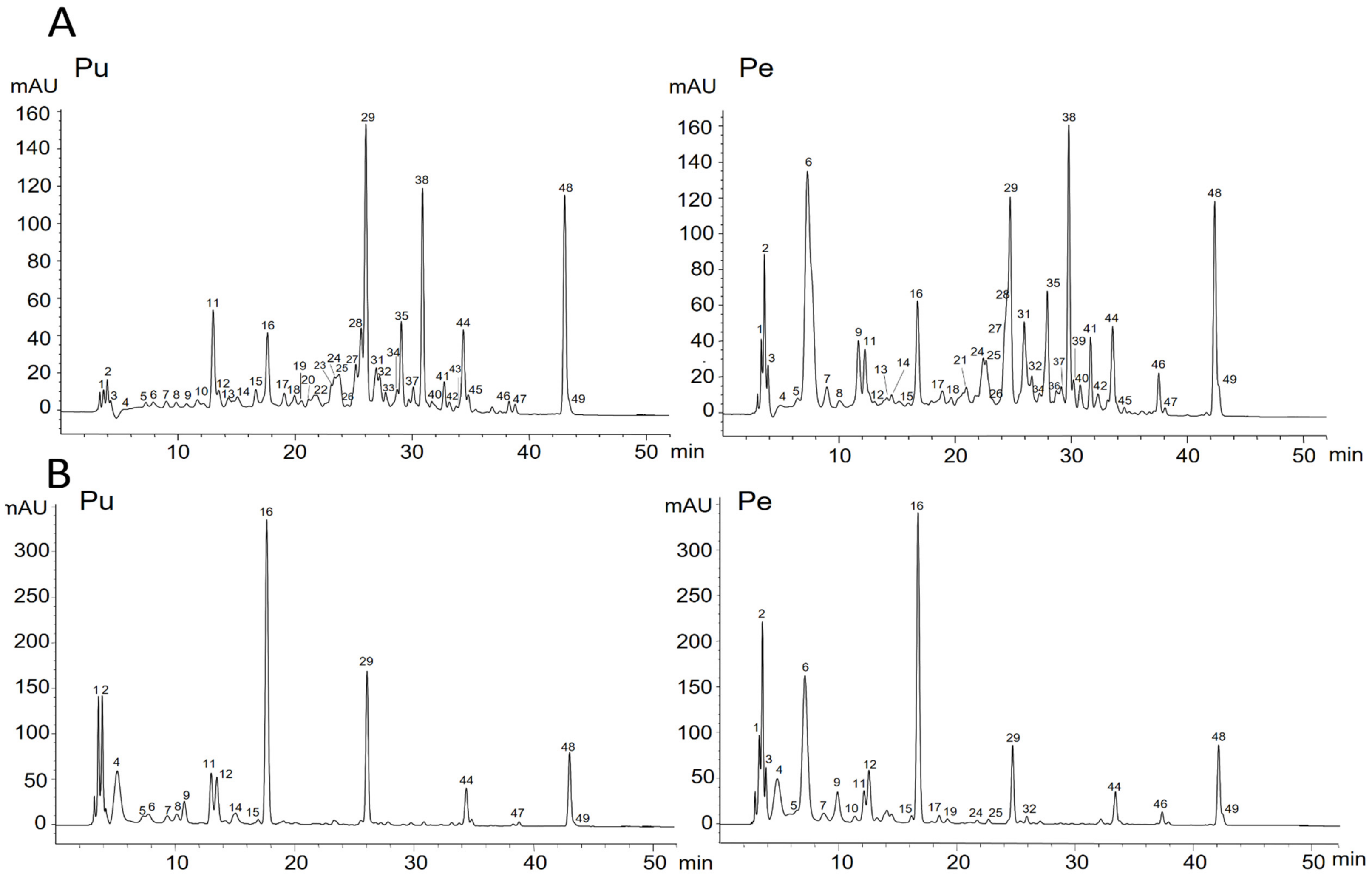

Figure S3. C_{30} reversed-phase chromatogram of carotenoids of direct (A) or saponified extracts (B) obtained from pulp (PU) and peel (PE) of papaya fruits (*Carica papaya*, L.) cv. Maradol, detected at 450 nm. Peak identities are showed in Table 2.

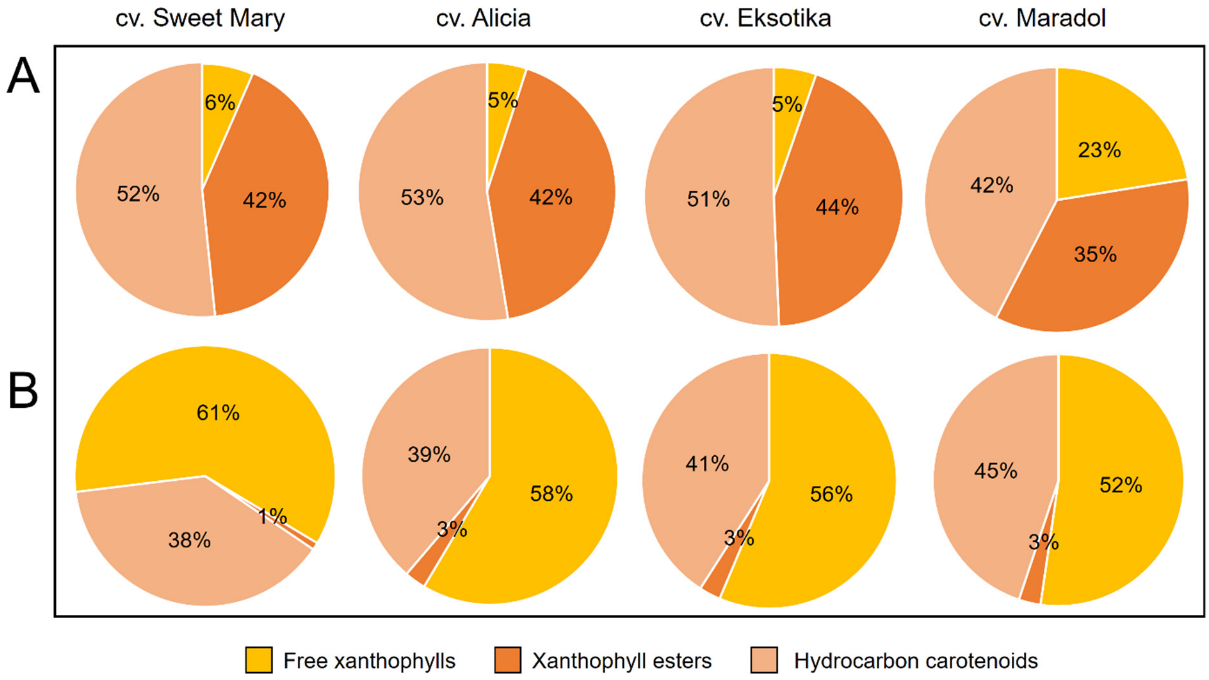

Figure S4. Carotenoid proportion (%) of free xanthophylls, hydrocarbon carotenoids and xanthophyll esters (A) in direct extracts (crude) and (B) in saponified extracts of papaya pulps of cv. Sweet Mary, cv. Alicia, cv. Eksotika and cv. Maradol.

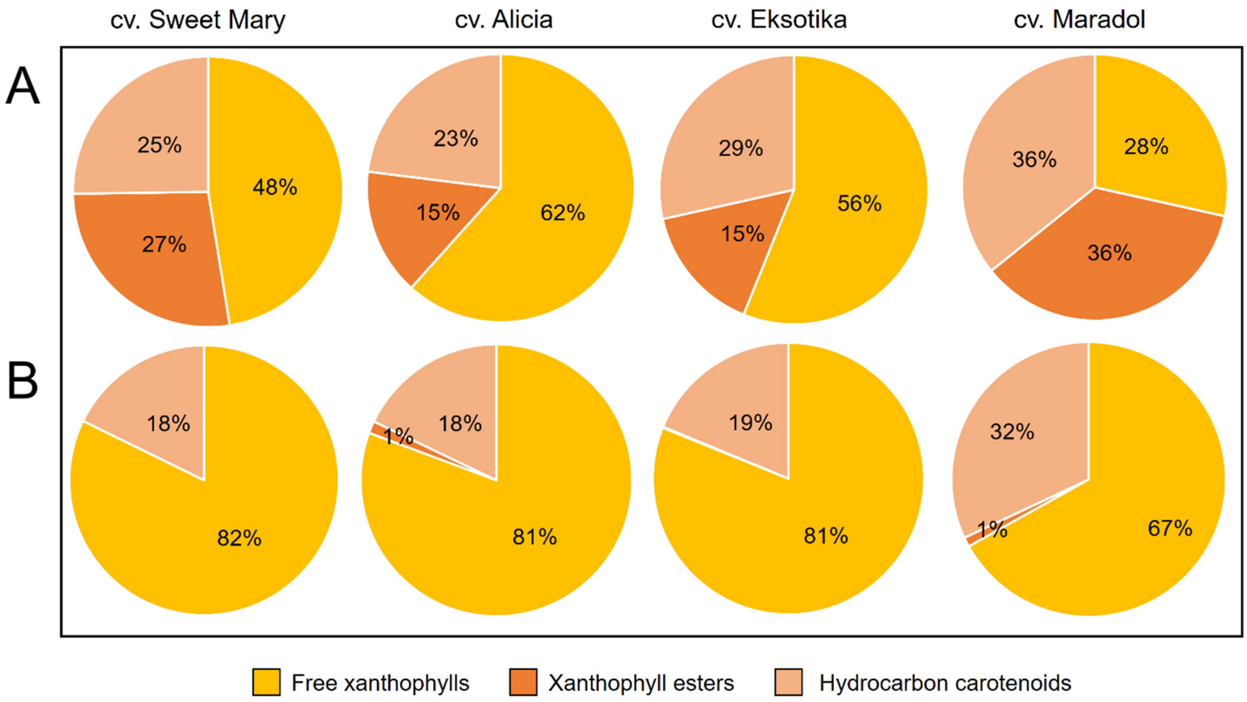

Figure S5. Carotenoid proportion (%) of free xanthophylls, hydrocarbon carotenoids and xanthophyll esters (A) in direct extracts (crude) and (B) in saponified extracts of papaya peels of cv. Sweet Mary, cv. Alicia, cv. Eksotika and cv. Maradol.

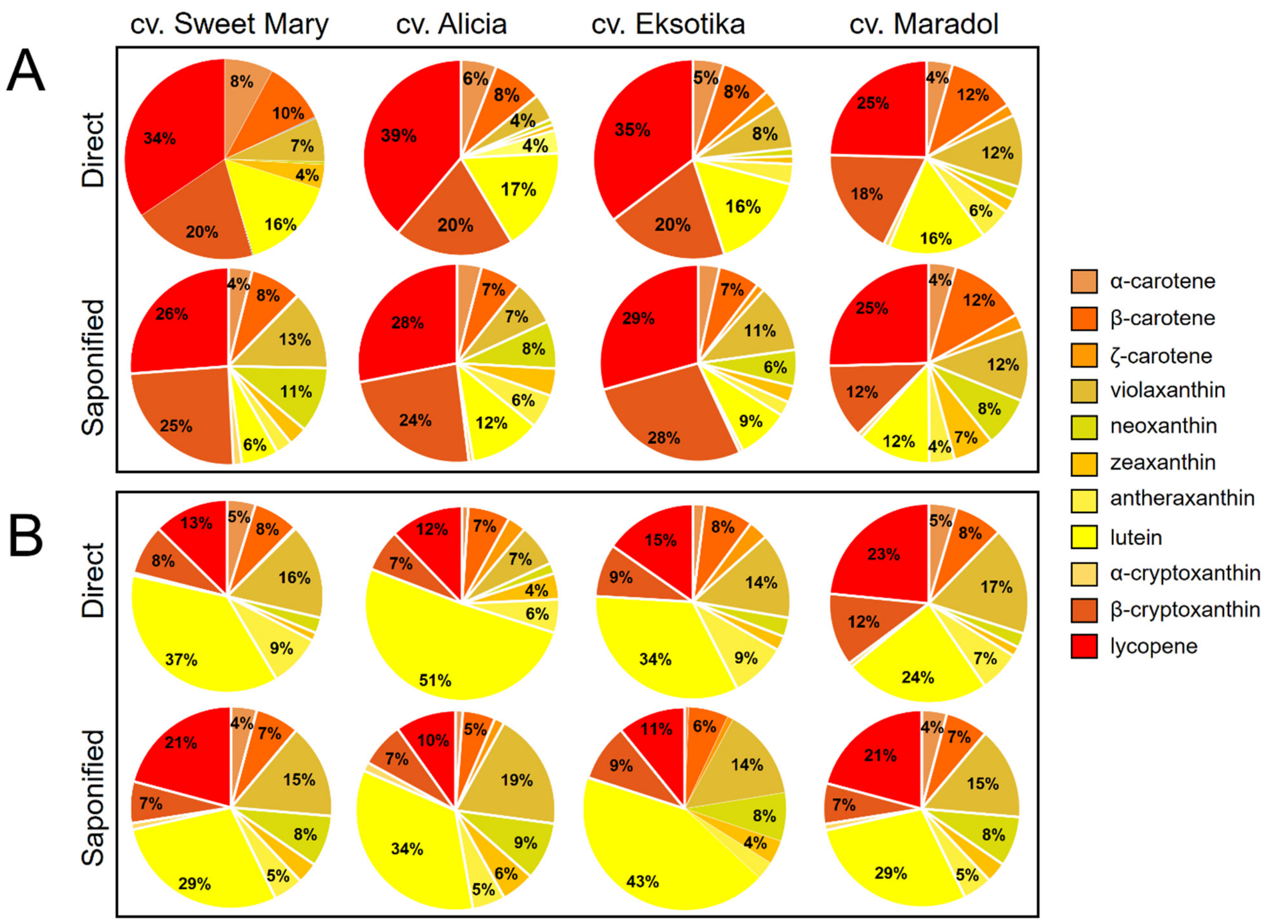

Figure S6. Carotenoid proportion (%) of carotenoid species in direct extracts (crude) and in saponified extracts of papaya (Carica papaya L.) (A) pulp and (B) peels of cv. Sweet Mary, cv. Alicia, cv. Eksotika and cv. Maradol.
